# Widespread seasonal gene expression reveals annual differences in human immunity and physiology

**DOI:** 10.1038/ncomms8000

**Published:** 2015-05-12

**Authors:** Xaquin Castro Dopico, Marina Evangelou, Ricardo C. Ferreira, Hui Guo, Marcin L. Pekalski, Deborah J. Smyth, Nicholas Cooper, Oliver S. Burren, Anthony J. Fulford, Branwen J. Hennig, Andrew M. Prentice, Anette-G. Ziegler, Ezio Bonifacio, Chris Wallace, John A. Todd

**Affiliations:** 1 JDRF/Wellcome Trust Diabetes and Inflammation Laboratory, Department of Medical Genetics, NIHR Cambridge Biomedical Research Centre, Cambridge Institute for Medical Research, University of Cambridge, Wellcome Trust/MRC Building, Cambridge Biomedical Campus, Cambridge CB2 0XY, UK; 2 MRC International Nutrition Group at MRC Unit The Gambia & London School of Hygiene & Tropical Medicine, Keppel Street, London WC1E 7HT, UK; 3 Institute of Diabetes Research, Helmholtz Zentrum München, Neuherberg, Forschergruppe Diabetes, Klinikum rechts der Isar, Technische Universität München, Ingolstaedter Landstr. 1, D 85764 Neuherberg, Germany; 4 CRTD—DFG Research Center for Regenerative Therapies Dresden, Paul Langerhans Institute Dresden, Medical Faculty, Technische Universität Dresden, Fetscherstrasse, 01307 Dresden, Germany; 5 MRC Biostatistics Unit, Cambridge Institute of Public Health, Forvie Site, Robinson Way, Cambridge Biomedical Campus, Cambridge CB2 0SR, UK

## Abstract

Seasonal variations are rarely considered a contributing component to human tissue function or health, although many diseases and physiological process display annual periodicities. Here we find more than 4,000 protein-coding mRNAs in white blood cells and adipose tissue to have seasonal expression profiles, with inverted patterns observed between Europe and Oceania. We also find the cellular composition of blood to vary by season, and these changes, which differ between the United Kingdom and The Gambia, could explain the gene expression periodicity. With regards to tissue function, the immune system has a profound pro-inflammatory transcriptomic profile during European winter, with increased levels of soluble IL-6 receptor and C-reactive protein, risk biomarkers for cardiovascular, psychiatric and autoimmune diseases that have peak incidences in winter. Circannual rhythms thus require further exploration as contributors to various aspects of human physiology and disease.

## Abstract (web summary)

Infectious and chronic diseases display seasonal patterns, yet seasonal changes in physiology are rarely thought to affect human health. Here the authors show seasonal variation in all major blood cells and in gene and protein expression in diverse tissues, suggesting a mechanism for seasonal disease proneness and physiology.

## Graphical abstract

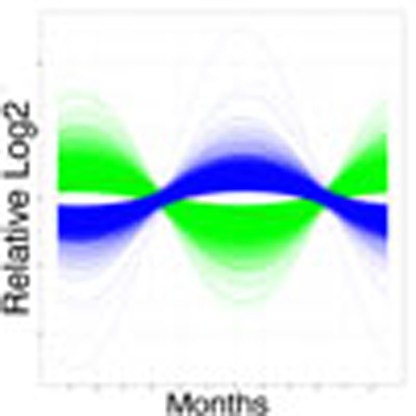

Periodic seasonal changes have influenced all life forms, as exemplified by seasonal physiology and behaviours across plant and animal species^1^,^2^,^3^. For example, reptile graft rejection^4^ and level of gonadal hormones in squirrel monkeys^5^ display seasonal variation. In humans, many complex polygenic diseases, including cardiovascular^6^,^7^, autoimmune^8^,^9^ and psychiatric illnesses^10^,^11^,^12^, have established seasonal patterns of incidence and disease activity. Infectious disease seasonality is well established in humans^13^, and it has been proposed that an inborn physiological rhythm underlies the seasonality of diagnoses of infectious diseases and their pathologies^14^, but direct evidence of such a system is lacking.

Various biological processes show seasonal variation in humans, including ones with important immunological roles, such as vitamin D metabolism^15^. The loss of skin pigmentation as humans migrated out of Africa to more temperate and colder zones to increase sunlight-driven vitamin D production is a major example of the evolutionary adaption of humans to different environments. Yet, how seasons might more broadly impact the underlying molecular details of human physiology is unknown. Along these lines, we hypothesized that the anti-inflammatory circadian transcription factor, *ARNTL* (BMAL1)^16^,^17^, would display seasonal gene expression differences as daylight entrains circadian rhythms in mammals^18^,^19^,^20^,^21^. Tissue-specific molecular clocks control a diverse range of cellular processes^22^,^23^, influencing the immune response^24^,^25^,^26^,^27^,^28^.

From ethnically and geographically diverse populations we analysed mRNA expression levels in peripheral blood mononuclear cells and adipose tissue biopsies, full blood count data, and the circulating levels of inflammatory protein biomarkers.

## Results

### Seasonal *ARNTL* expression in the immune system

**Figure 1:**
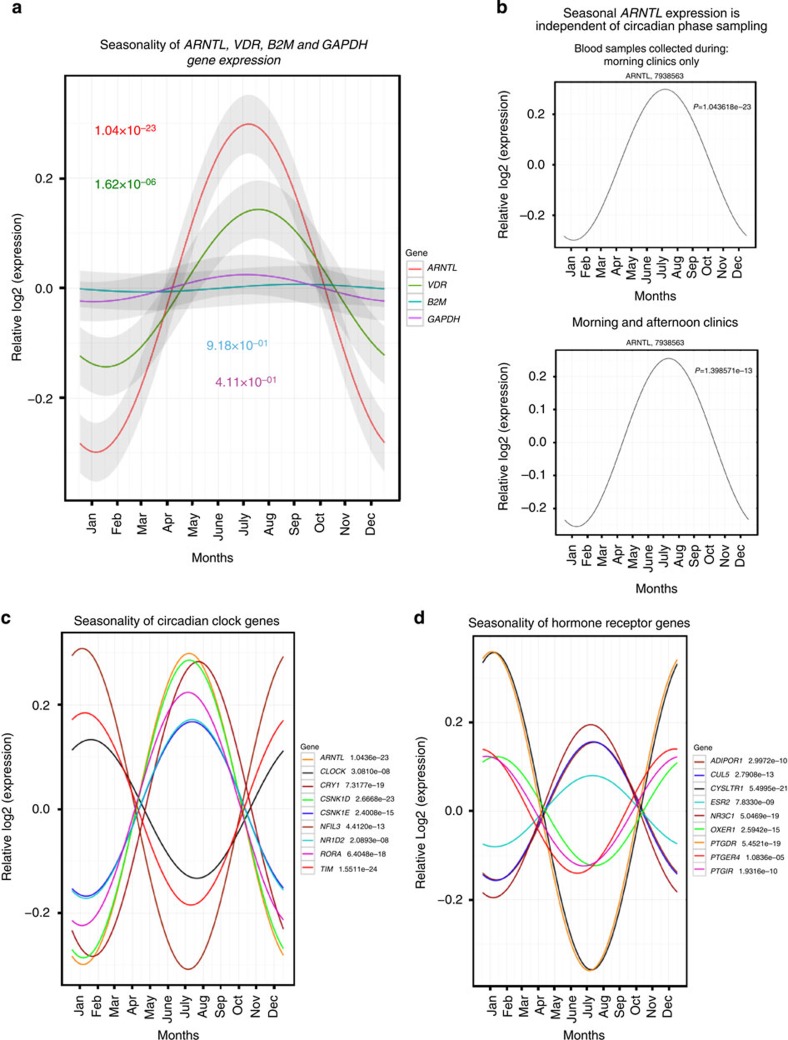
Seasonal mRNA expression in the peripheral human immune system. Relative expression profiles of seasonal genes (fitted values of the cosinor model). (**a**) *ARNTL* expression was increased in the summer months of June, July and August (ANOVA, 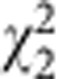, *P*=1.04 × 10^−23^), compared with the winter months of November through February (1.5097-fold difference between February and August (*n*=109 individuals). Similarly, the nuclear vitamin D receptor (*VDR*) shows peak expression in June through August (ANOVA, 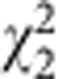, *P* = 1.62 × 10^−06^). The housekeeping genes, *B2M* and *GAPDH*, did not have seasonal expression profiles. (**b**) Seasonal *ARNTL* expression in PBMCs independent of the circadian phase. Similar seasonal *ARNTL* expression profiles were observed regardless of whether blood samples were collected during morning (BABYDIET, *n*=109 individuals) or afternoon clinic visits (T1D cohort, *n=*236 individuals). (**c**) In the BABYDIET data set, nine known components of the circadian clock had seasonal expression profiles in the peripheral immune system, as did certain hormone, leukotriene and prostaglandin receptors. (**d**) The receptors for the anti-inflammatory glucocorticoids (*NR3C1*) and the pro-inflammatory prostaglandins (*PTGDR*, *PTGIR* and *PTGER4*) and leukotrienes (*CYSLTR1*) had opposing seasonal expression profiles.

We first analysed *ARNTL* expression in peripheral blood mononuclear cells (PBMCs) from children (454 samples from 109 individuals) enroled into the BABYDIET cohort from Germany^29^ (Supplementary Table 1). *ARNTL* mRNA showed seasonal variation in expression (ANOVA, 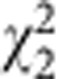, *P*=1.04 × 10^−23^), peaking in the summer months of June, July and August (Fig. 1a). The difference between the winter low and summer high in *ARNTL* expression was 1.5097-fold. Vitamin D receptor (*VDR*) expression was also higher in the summer months (Fig. 1a). The housekeeping genes, *B2M* and *GAPDH*, often used as standards in gene expression analyses, did not show seasonal variation (Fig. 1a). *ARNTL* showed the same seasonal expression profile independently of whether blood was drawn during morning or afternoon clinic visits (Fig. 1b), suggesting that diurnal oscillations are not responsible for the seasonal differences in *ARNTL* expression.

We then sought evidence for seasonality in known components of the circadian clock. Seasonal variation was found in 9 of the 16 clock genes tested: *ARNTL*, *CLOCK*, *CRY1*, *CSNK1D*, *CSNK1E*, *NR1D2*, *RORA*, *TIMELESS*^30^ and *NFIL3* (which controls diurnal Th17 cell development in mice^31^) (Fig. 1c). Seven genes (*CRY2*, *PER3*, *RORB*, *NPAS2*, *PER1*, *PER2* and *NR1D1*) did not show evidence for seasonal effects (Supplementary Table 3). Novel components of the human circadian clock, as well as clock-targeted genes and pathways, are likely to be present among the genes whose expression correlated with *ARNTL* (Supplementary Table 2). Interestingly, the glucocorticoid receptor (*NR3C1*) had a strong positive correlation with *ARNTL* (Spearman *ρ*=0.819), with lowest expression in the winter (ANOVA, 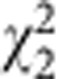, *P*=5.05 × 10^−19^) (Fig. 1d). Glucocorticoids have anti-inflammatory properties^32^ and SCN-controlled hormones are thought to be essential molecules for maintaining the synchronicity of peripheral biological clocks^33^. In contrast to *NR3C1*, receptors for the prostaglandins (*PTGDR*, *PTGIR* and *PTGER4*), leukotrienes (*CYSLTR1*) and oxoeicosanoids (*OXER1*) were more highly expressed in the winter in Germany. Receptors for adiponectin (*ADIPOR1*), estradiol (*ESR2*) and antidiuretic hormone (*CUL5*) were more highly expressed in the summer (Fig. 1d). Other hormone receptors did not show any seasonal variation in this data set.

### Widespread seasonal gene expression in the immune system

**Figure 2:**
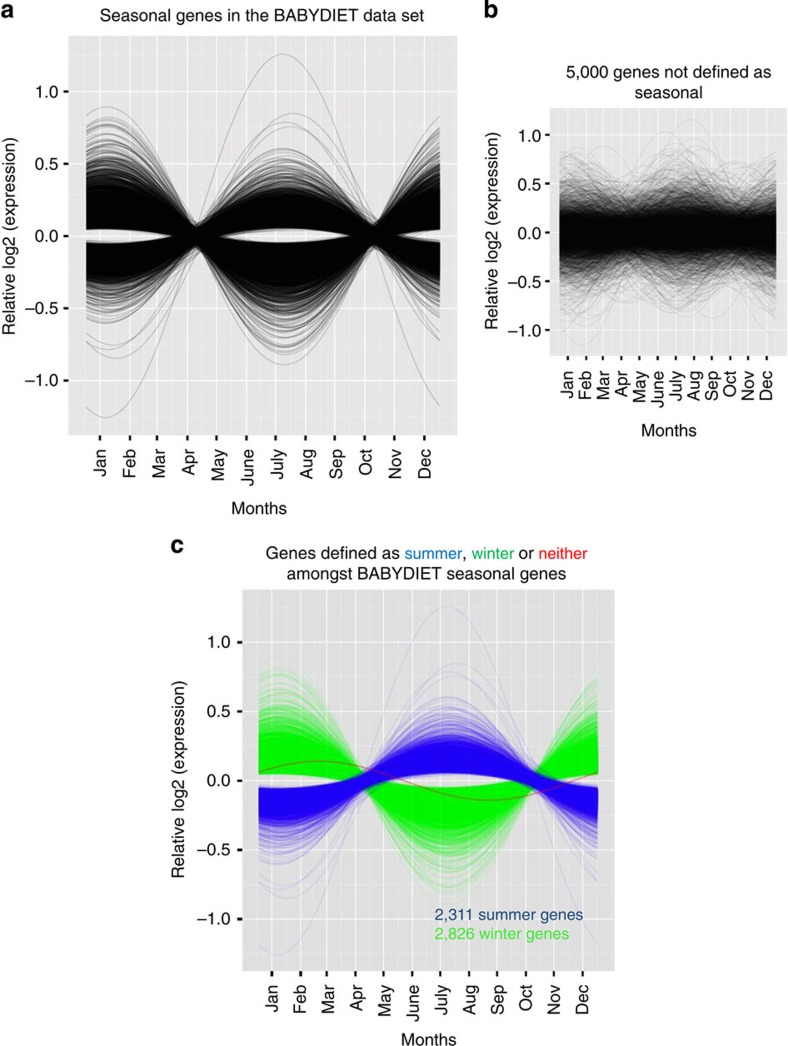
Widespread seasonal mRNA expression in the human immune system. Relative expression profiles of seasonal genes (fitted values of the cosinor model). (**a**) A total of 5,136 genes (∼23% of the protein-coding genome) were identified as having seasonal variation in expression (genome-wide significance, *P*≤1.52 × 10^−06^) in the BABYDIET PBMC data set. (**b**) Five thousand randomly selected genes not identified as seasonal are shown as a comparison. (**c**) Two anti-phasic patterns of gene expression were observed among seasonal genes. We defined the majority of seasonal genes as being either winter- (green) or summer-expressed (blue). In BABYDIET, 2,311 genes were increased in expression in the summer and 2,826 were increased in the winter. One of the seasonal probes did not fall into our definition of summer and winter, shown as a red line.

Strikingly, we found ∼23% of the genome (5,136 unique genes out of 22,822 genes tested) to show significant seasonal differences in expression in the BABYDIET data set (Fig. 2a and Supplementary Table 3). Among the seasonal genes, two distinct anti-phasic patterns of gene expression were evident: 2,311 genes (2,922 unique probes) had increased expression in the summer (defined as June, July and August, mean fold change=1.2572) while 2,826 genes (3,436 unique probes) were upregulated in the winter (defined as December, January, February, mean fold change =1.3150) (Fig. 2c, Supplementary Fig. 1), demonstrating that different transcriptional landscapes are present in the peripheral immune system during different seasons.

The daily variables of mean ambient temperature and mean sunlight hours both served as linear predictors of seasonality (Supplementary Fig. 2), suggestive of human environmental adaptation.

**Figure 3:**
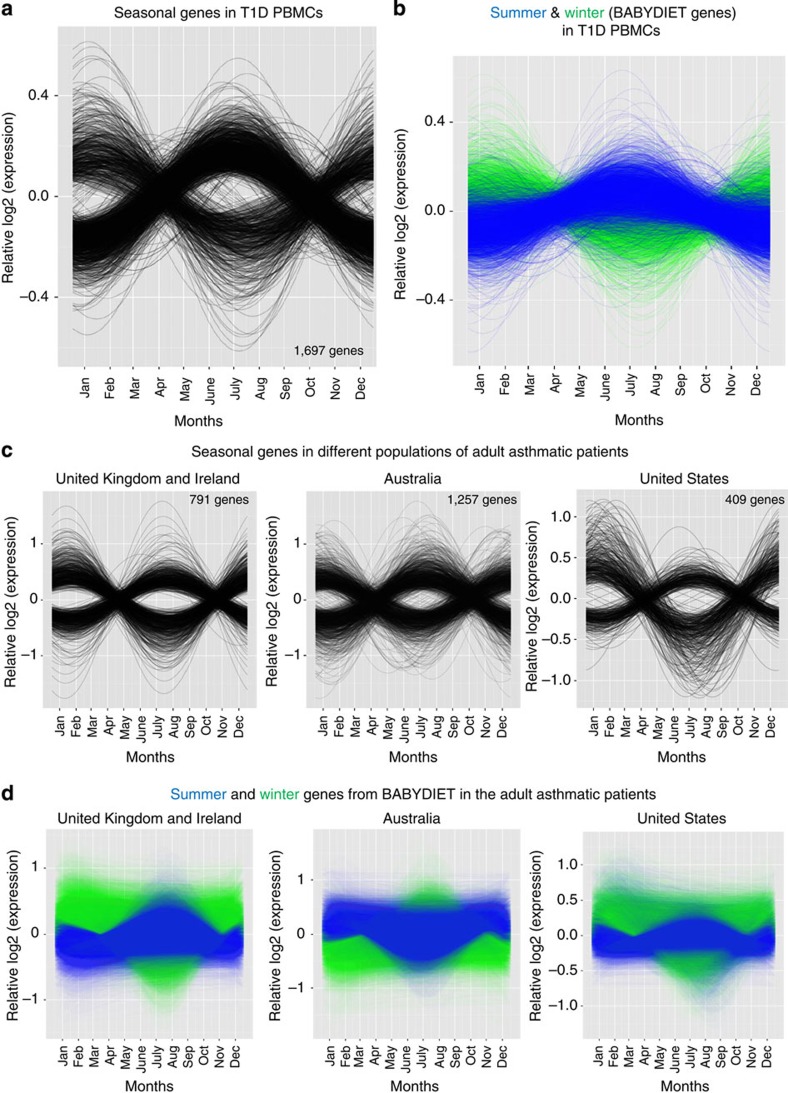
Seasonal gene expression in geographically distinct cohorts. (**a**) Seasonality was also observed in PBMCs collected from T1D patients in the United Kingdom (*n*=236 individuals). A total of 1,697 genes were seasonal in this data set. (**b**) The previously defined summer and winter genes from the BABYDIET data set maintained their seasonal expression patterns in the T1D samples. (**c**) PBMCs from asthmatic patients collected from different countries also showed seasonal gene expression. In the United Kingdom/Ireland (*n*=26 asthmatic individuals; 85 PBMC samples), 791 genes were seasonal, while 1,257 and 409 genes were seasonal in Australia (*n*=26 individuals; 85 samples) and United States (*n*=37 individuals; 123 samples), respectively. (**d**) Summer and winter BABYDIET genes maintained their seasonal expression patterns in the asthmatic PBMC samples, with their patterns inverted in Australia.

We replicated the observation in two independent data sets. First, in a collection of PBMCs isolated from autoimmune type 1 diabetes (T1D) patients (236 samples) from the United Kingdom, 1,697 genes were found to exhibit seasonal expression (Fig. 3a and Supplementary Table 4). The majority of seasonally associated transcripts could again be identified as having summer or winter expression profiles, with seasonal patterns matching those identified in the BABYDIET data set (Fig. 3b). This data set demonstrated seasonal gene expression in adults, adding to the observations made in samples from children (Supplementary Fig. 3 and Fig. 2).

Secondly, we analysed gene expression data from a collection of PBMCs from adult (18–83 years old, mean age 45) asthmatic individuals from diverse ethnic groups across Australia, United Kingdom/Ireland, United States and Iceland^34^. We separated the entire cohort into distinct geographical locations and observed seasonal gene expression in each (Fig. 3c and Supplementary Tables 5–8). Seasonal genes identified in the BABYDIET cohort maintained their seasonal tropisms in the asthmatic patients (Fig. 3d). Most interestingly, in the Australian data set, the previously defined summer genes (increased in expression during the Northern hemisphere summer) were more highly expressed during the Southern hemisphere summer, spanning December, January and February (Fig. 3d); clearly illustrated, for example, by *ARNTL* expression (Supplementary Fig. 6B). The pattern of seasonal gene expression in samples from Iceland was unique (Supplementary Fig. 4).

Seasonal gene expression was not altered in samples from children with self-reported infections^35^ (Supplementary Fig. 5), and the type-I interferon response gene, *SIGLEC1*^35^, was not seasonal. Finally, recruitment into the asthma cohort was dependent on participants being free from infectious diseases^34^. Nevertheless, the relationship between seasonal infections and diseases, and these seasonal gene expression patterns, remains to be fully described.

### Common seasonal genes in the immune system

One hundred and forty-seven genes showed common seasonality in the BABYDIET, T1D, Australia, USA and UK/Ireland datasets (Supplementary Table 9). These 147 genes had similar seasonal expression patterns in each cohort (Supplementary Fig. 6). Notably, in the Icelandic cohort, the common seasonal genes did not share the same expression pattern (Supplementary Fig. 4). This could be due to near-24-h daylight during summer if seasonal human physiology is regulated by changes in the annual photoperiod^36^. *ARNTL* was found to be a common seasonal gene (Fisher's method, 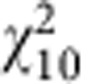, *P*=6.73 × 10^−57^), with increased summer expression in each PBMC data set, except Iceland. The gene with the strongest seasonal profile common to all data sets (excluding Iceland) was *C14orf159* (winter expressed, Fisher's method, 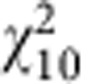, *P*=3.93 × 10^−66^). The mitochondrial protein, UPF0317, encoded by *C14orf159* (whose expression is regulated by oestrogen receptor alpha^37^) is highly conserved in chordates, although its function in humans is largely unknown.

### Seasonal cellular remodelling of the human immune system

As PBMCs represent several specialized haematopoietic lineages, we sought to determine whether seasonal gene expression resulted from annual changes in the cellular composition of blood.

**Figure 4:**
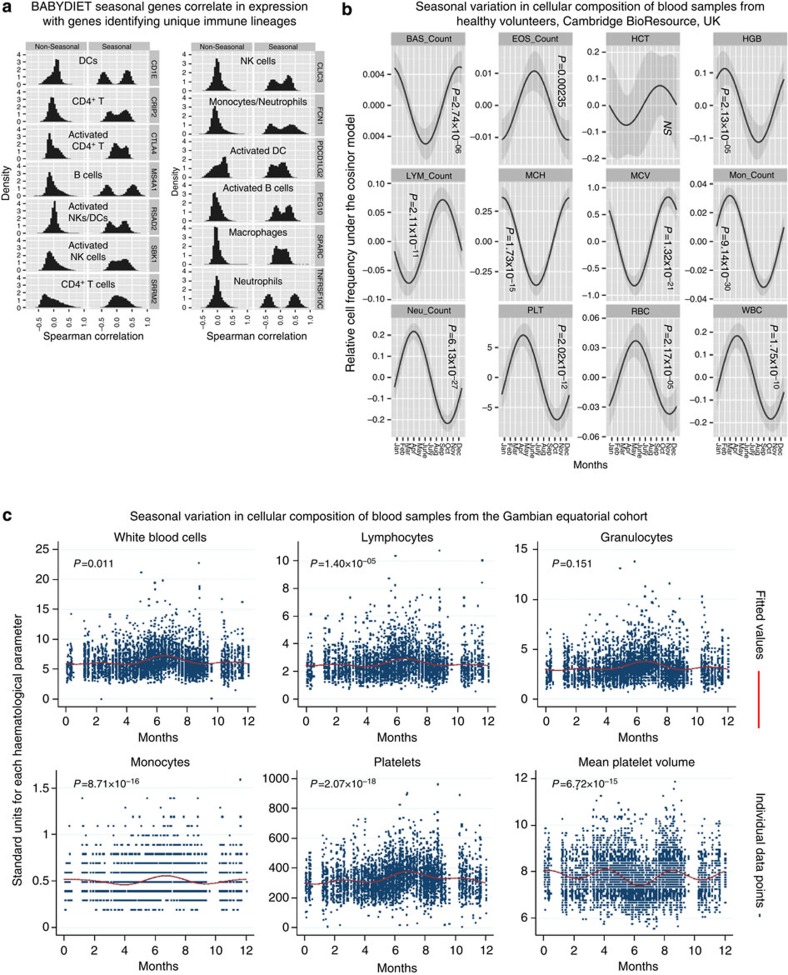
Seasonal changes in the cellular composition of human peripheral blood. (**a**) Expression levels of 13 genes that have been used to identify different blood cell types among total PBMCs were strongly correlated (positively and negatively) with seasonal genes identified in the BABYDIET data set. In comparison, non-seasonal genes were less correlated with these marker genes, although exceptions exist: CTLA-4 expression also correlations with non-seasonal genes. (**b**) Indeed, by analysing full blood count data obtained from 7,343 healthy adult donors enroled in the Cambridge BioResource, we found the cellular composition, and other haematological parameters of blood to vary by season. HCT was the only response that did not show seasonal variation. (**c**) Distinct seasonal variation in cell counts was observed in a cohort of 4,200 healthy adults and children from The Gambia. EOS, eosinophils; LYM, lymphocytes, NEU, neutrophils, PLT, platelets; RBC, red blood cells; WBC, total white blood cells; BAS, basophils; HGB, haemoglobin; MCH, mean corpuscular haemoglobin; MCV, mean corpuscular volume; MON, monocytes; HCT, haematocrit.

In support of this, we found the expression of seasonal genes to correlate strongly with the expression of 13 genes known to mark different immune cell types present in PBMCs^38^ (Fig. 4a). Furthermore, by analysing full blood count (FBC) data from 7,343 healthy adult donors enroled in the Cambridge BioResource (United Kingdom), we found the total number of white blood cells (ANOVA, F-test, *P*=1.75 × 10^−10^), lymphocytes (ANOVA, F-test, *P*=2.11 × 10^−11^), monocytes (ANOVA, F-test, *P*=9.14 × 10^−30^), basophils (ANOVA, F-test, *P*=2.74 × 10^−6^), eosinophils (ANOVA, F-test, *P*=0.00235), neutrophils (ANOVA, F-test, *P*=6.13 × 10^−27^) and platelets (ANOVA, F-test, *P*=2.02 × 10^−12^) to exhibit seasonality in the peripheral circulation, as did the mean corpuscular volume (MCV) (ANOVA, F-test, *P*=1.32 × 10^−21^) and mean corpuscular haemoglobin (MCH) (ANOVA, F-test, *P*=1.73 × 10^−15^) of erythrocytes (Fig. 4b). Our results are in agreement with a study that reported seasonal red blood cell and platelet gene expression^39^.

In a more equatorial cohort, comprising 4,200 healthy individuals from The Gambia (West Africa), we observed seasonal variation in the number of total white blood cells (F-test, *P*=0.011), lymphocytes (F-test, *P*=1.40 × 10^−05^), monocytes (F-test, *P*=8.71 × 10^−16^) and platelets (F-test, *P*=2.07 × 10^−18^) (Fig. 4c), but not granulocytes. We also observed striking seasonal variation in red blood cell numbers (F-test, *P*=8.43 × 10^−30^) and their mean corpuscular haemoglobin (F-test, *P*=4.07 × 10^−30^) (Supplementary Fig. 7). The seasonal patterns in The Gambia were completely distinct to those observed in the UK cohort. In The Gambian cohort, the numbers of all seasonal cell types peaked during the rainy season (as previously reported for leukocytes^40^), June through October, during which time the immune system faces different pathogenic challenges, such as an increased infectious disease burden, including malaria^41^.

### Seasonal differences in human immunity

**Figure 5:**
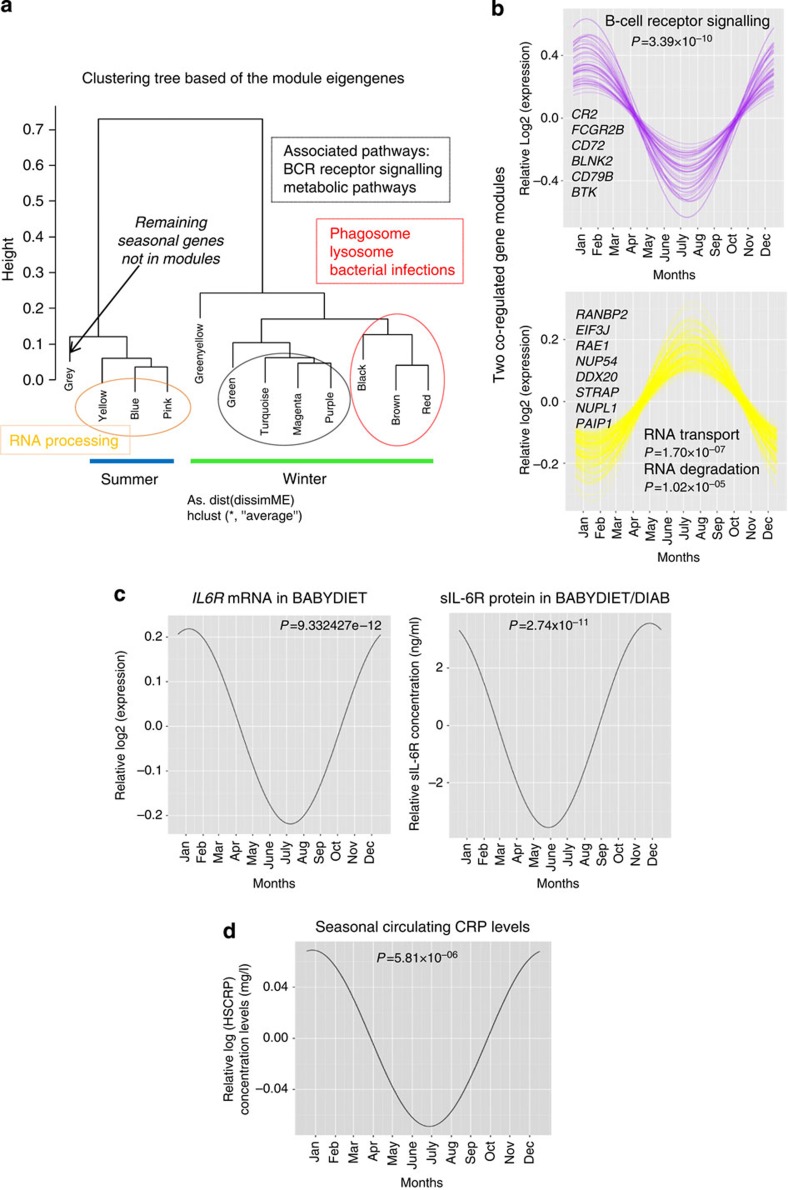
Inflammatory responses predominate the immune system in Europe. (**a**) Co-regulated seasonal gene modules were generated to analyse differences in immune function by season: eight winter modules and three summer modules were generated. (**b**) Two modules of seasonally co-regulated genes from the BABYDIET data set are shown as examples. A module consisting of genes involved in B-cell receptor signalling, (including *CR2*, *BLNK*, *BTK*, *FCGR2B*, *CD72*, *CD79B*) was more highly expressed in the winter, as was a module associated with metabolic processes. In contrast, a RNA-processing module (containing *RANBP2*, *EIF3J*, *RAE1*, *NUP54*, *DDX20*, *STRAP*, *NUPL1*, *PAIP1*) was more highly expressed in the summer. (**c**) IL6R mRNA expression was increased in the winter, in BABYDIET samples (ANOVA, 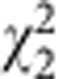, *P*=9.33 × 10^−12^), as was observed for the circulating level of sIL-6R protein in the serum of BABYDIET/DIAB children (ANOVA, 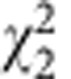, *P*=2.74 × 10^−11^). (**d**) The circulating levels of C-reactive protein displayed seasonal variation in a cohort of 3,412 donors diagnosed as hypertensive but not conventionally dyslipidemic. ASCOT enrolled participants in Ireland, Denmark, Finland, Iceland, Norway, Sweden and the UK (two measurements per donor), with increased levels present during winter HSCRP - high sensitivity C-reactive protein.

To address whether immunological function varies seasonally, as suggested by the transcriptomic and cell count data, we generated modules of co-regulated seasonal mRNAs identified in the BABYDIET data set (Fig. 5a and Supplementary Fig. 8 and Supplementary Table 12). Among the seven winter-expressed modules we identified, we found pro-inflammatory processes to be more frequent, compared with the identified summer-expressed modules. B-cell receptor (BCR) signalling (Hypergeometric test, *P*=3.39 × 10^−10^), FcR-gamma-associated processes (Hypergeometric test, *P*=4.45 × 10^−6^), lysosomes (Hypergeometric test, *P*=2.96 × 10^−5^), chemokine signalling (Hypergeometric test, *P*=3.56 × 10^−5^) and phagosomes (Hypergeometric test, *P*=5.97 × 10^−5^) were all strongly associated with winter-expressed modules. In contrast, RNA transport (Hypergeometric test, *P*=1.70 × 10^−7^), RNA degradation (Hypergeometric test, *P*=1.02x10^−5^), ubiquitin-mediated proteolysis (Hypergeometric test, *P*=0.0002), circadian rhythms in mammals (Hypergeometric test, *P*=0.0011) and splicosome (Hypergeometric test, *P*=0.0014) were the most-associated pathways with summer-expressed modules, suggesting that a more inflammatory status of the immune system predominates in winter (Fig. 5b and Supplementary Fig. 8).

In further support of this, we found the concentration of sIL-6R protein to be increased in winter in samples from BABYDIET and BABYDIAB (a related collection^42^; ANOVA, 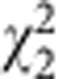, *P*=2.74 × 10^−11^), in complete agreement with the increased winter expression of *IL6R* mRNA in BABYDIET samples (ANOVA, 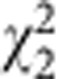, *P*=9.33x10^−12^) (Fig. 5c). sIL-6R is an important orchestrator of leukocyte recruitment^43^ and trans-presents IL-6 to cells expressing gp130 in the absence of the cell-surface IL-6R^44^, endowing IL-6 with a broader spectrum of influence. Indeed, a coding variant in *IL6R* that alters circulating sIL-6R concentration is associated with impaired IL-6 signalling and the protection from cardiovascular disease, rheumatoid arthritis and T1D^45^. Interestingly, early-stage inflammation in rheumatoid arthritis (a disease treated with anti-IL-6 receptor reagents^46^) has been shown to either resolve or progress to erosive disease, and a predictor of this outcome is the season when disease symptoms first present^47^. T1D also has seasonal trends in diagnoses^9^ and autoantibody positivity^48^, suggesting that seasonal environments impact on autoimmune disease pathologies. Furthermore, we found the circulating level of the acute-phase complement activator, C-reactive protein^49^, to be increased during winter months (Fig. 5d).

These gene expression data also suggest that the quality of a vaccine response may be influenced by season. We found the expression of *TLR7* (ANOVA, 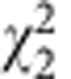, *P*=4.22 × 10^−16^), *TLR8* (ANOVA, 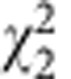, *P*=3.70 × 10^−09^) and *DDX58* (encoding the viral RNA receptor RIG-I, ANOVA, 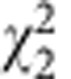, *P*=9.65 × 10^−20^) to have increased in expression in winter months in the BABYDIET data set: the increased expression of these genes correlated with protective immunity in response to the Yellow Fever vaccine (YF-17D)^50^. *TNFRSF17* also showed seasonal variation in the BABYDIET data set (ANOVA, 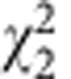, *P*=1.30 × 10^−12^), and its induction is shown to be predictive of an antibody response after trivalent influenza vaccine^51^,^52^. Furthermore, *OAS1* (ANOVA, 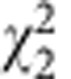, *P*=1.43 × 10^−20^), *OAS2* (ANOVA, 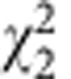, *P*=2.85 × 10^−16^), *STAT2* (ANOVA, 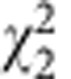, *P*=1.76 × 10^−18^), *POU2AF1* (ANOVA, 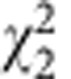, *P*=3.27 × 10^−15^) and *CD27* (ANOVA, 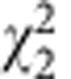, *P*=3.29 × 10^−16^) were also seasonal and their expression in PBMCs was correlated with increased anti-DT IgG responses after the meningococcal vaccine (MCV4)^51^. The antibody responses to rabies, typhoid and pneumococcal vaccines are influenced by the month of vaccine administration^40^.

### Seasonal gene expression in subcutaneous adipose tissue

Given the remarkable seasonality of the peripheral immune system and the correlations we found with multiple health-associated phenotypes, we anticipated that tissues throughout the body would display extensive seasonality of gene expression. We were able to analyse gene expression data from a collection of subcutaneous adipose tissue samples obtained from 825 healthy female donors enroled in the TwinsUK cohort^53^.

**Figure 6:**
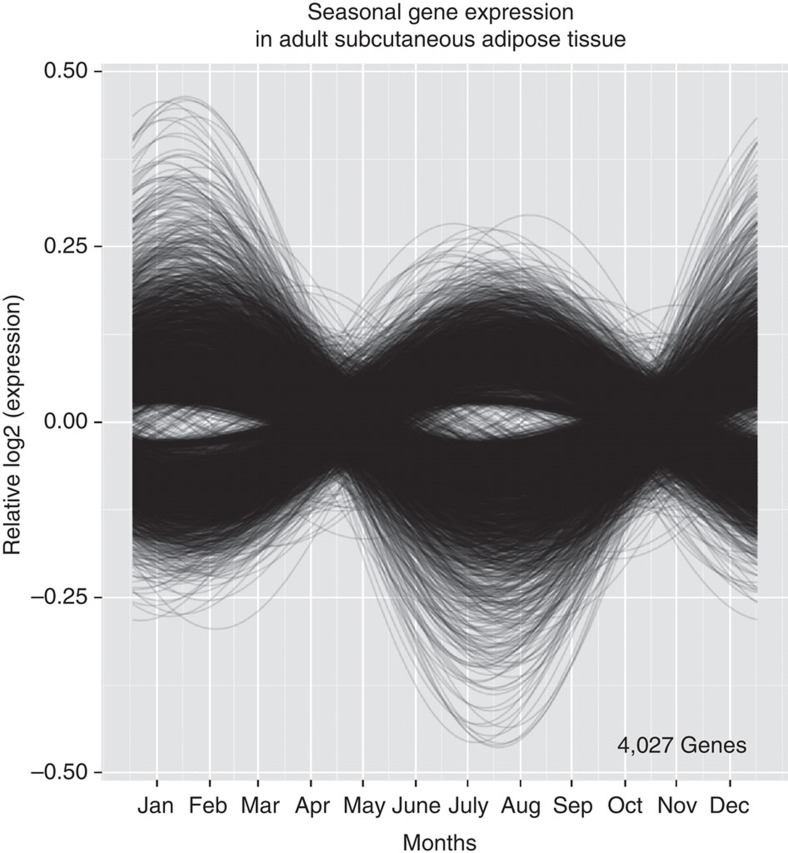
Seasonal gene expression in subcutaneous adipose tissue. In a collection of 856 female adult donors from the United Kingdom, 4,027 genes were found to be seasonal in adipose tissue. As observed in PBMCs, two distinct anti-phasic profiles were present.

We found 4,027 genes to be seasonally expressed (Fig. 6, Supplementary Table 13), including *IL6ST* (gp130) (ANOVA, 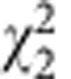, *P*=2.55 × 10^−8^) and *IL6R* (ANOVA, 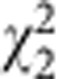, *P*=1.49 × 10^−8^): adipose tissue can produce IL-6^54^. One thousand, two hundred and thirteen genes were common to both adipose tissue and BABYDIET data sets (Supplementary Table 14), suggesting that common genetic mechanisms regulate seasonality.

In adipose tissue, as in PBMCs, metabolic pathways were among the most associated seasonal pathways (Supplementary Fig. 9). Such seasonal metabolic programmes may have been selected for due to annual differences in temperature and diet. Adipose tissue seasonality has important implications for immunology, obesity and metabolic disease research; for example, *PPARG*, targeted by thiazolidinediones as a current treatment of type 2 diabetes, was found to be seasonal in adipose tissue (ANOVA, 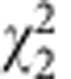, *P*=3.75 × 10^−9^).

## Discussion

Ecological changes alter the types and dynamics of inter- and intra-organism biological processes, and it follows that such changes will be manifested as seasonal transcriptional signatures within the immune systems of different organisms, which adapt to their environment. Studies of the function, dynamics and variability of the immune system are undergoing a long-awaited renaissance partly owing to the development and application of new phenotyping technologies^55^,^56^. Nevertheless, to date, no study to our knowledge has taken into account the variability we have observed in the immune system according to season, which could, for example, increase the differences in some immune phenotypes between twins or other family members if blood samples were collected at different times of year. We observed seasonal differences in expression across a large number of genes in mixed populations of human peripheral white blood cells from geographically and ethnically diverse locations, and, remarkably, seasonal genes displayed opposing patterns in the Southern and Northern hemispheres. Fewer seasonal genes were identified in Icelandic donors, and common seasonal genes had a less similar seasonal pattern in this data set. If a seasonal photoperiodic clock exists in humans, the impact of living at higher latitudes requires further exploration.

These periodically changing transcriptional landscapes in PBMCs, which appear to be predominantly driven by annual changes in the cellular composition of blood, are likely to influence various aspects of the human immune response. Indeed, the increased winter expression of co-regulated pro-inflammatory gene modules, the functionally important increased concentration of sIL-6R and CRP in the blood, and the observation that a loss of BMAL1 (*ARNLT* was reduced in winter) promotes inflammation in mice^28^, strongly suggests that the immune system is more pro-inflammatory in Europeans during the northern hemisphere winter. In mice, *Arntl*-BMAL1 controls the diurnal variation of circulating and tissue-resident inflammatory monocyte numbers^28^, although how *ARNTL* controls human immune function is not known. We note that, in Europeans, total monocyte numbers in blood are increased during winter, when *ARNTL* expression is the lowest. Notably, acute-phase proteins including CRP are induced by IL-6, which can be produced by macrophages and adipocytes^54^. This entire network could be a major factor in the higher frequency of cardiovascular disease-associated deaths in winter^6^, when increased risk is associated with excessive inflammation, IL-6 and monocytes. Furthermore, increased IL-6 signalling is associated with increased risk of rheumatoid arthritis and type 1 diabetes^45^, which peaks in incidence during the European winter. Increased IL-6 signalling and elevated CRP levels have also been associated with neuropsychiatric symptoms in children and adults^57^,^58^. Thus, modulation of IL-6 signalling according to season could be considered as a therapeutic strategy in various disease contexts. Whether a seasonal human immune system contributes to host-mediated pathology and morbidity after infection^59^ remains to be determined, but the correlations we report suggest this might be the case.

The breadth and functional characteristics of the seasonal gene expression we observed suggest that it has been evolutionarily selected for. During European winters, the thresholds required to trigger an immune response may be lower as a direct consequence of our co-evolution with infectious organisms and increased inter-species competition during winter, especially as humans migrated out of Africa to colder, more seasonally pronounced latitudes. In our European cohorts, winter was associated with increased monocytes and inflammation, while FBC data from the more-equatorial Gambian cohort exhibited distinct seasonal variation in cell numbers. In this data set, seasonal peaks in cell numbers correlated with the rainy season (June to October), during which time the infectious disease burden is at its highest levels.

Regardless of any particular causal factor driving these differences, which are likely many, our results demonstrate that different human populations independently vary the cellular composition of their immune system by season, suggestive of distinct environmental adaptations. Furthermore, although our data suggest that cell-type numbers contribute the majority of seasonal gene expression in PBMCs, future studies of seasonal phenotypic differences within purified immune cell subsets are likely to reveal an additional layer of complexity in the human immune system.

The origin and likely diverse mechanisms maintaining seasonal variation remain to be established: daylight and ambient temperature are candidate environmental cues that could co-ordinate seasonal hormonal phenotypes and cell-fate decisions in haematopoietic and stem cells. Indeed, diurnal entrainment of the human circadian clock requires daylight changes, demonstrating that humans sense and process photoperiodic cues to co-ordinate physiology.

The environmental perturbation of our molecular clocks is thought to be deleterious to health^60^, which may help explaining the increasing complex disease burden in industrialized countries^61^ and populations at extreme latitudes^9^, where clock dysregulation or chronodisruption may be more frequent^62^. In seasonally-breeding mammals, circadian melatonin production cues reproduction in response to changes in the annual photoperiod^63^. In the arctic mammal, *Rangifer tarandus*, daily melatonin rhythms are acutely responsive to the night-day phase but not the circadian phase^64^, demonstrating species-specific adaptation to the unique night-day cycles present at extreme latitudes: the ability of humans to properly function in such environments is not well understood. Furthermore, a circannual molecular clock was recently shown to control seasonal reproduction in hamsters, independently of melatonin and sex steroids, yet using the same neuroendocrine reproductive pathway^65^. Human genetic variation in the *ARNTL* gene region has been associated with age of menarche^66^,^67^, which is also seasonal.

The widespread seasonal gene expression observed in subcutaneous adipose demonstrates seasonality across different human tissues.

Regardless of the mechanisms causing and maintaining these and other seasonal variations, our results provide a plausible mechanism to explain part of the seasonality of human disease. These data provide a fundamental shift in how we conceptualize immunity in humans, and we propose that seasonal changes be more broadly considered as major determinants of human physiology.

## Methods

### Study subjects and human samples

All samples and information were collected with written and signed informed consent. One hundred and nine children genetically predisposed to T1D were enrolled in the BABYDIET study. The BABYDIET study is an intensively monitored dietary intervention study testing the potential effect of delayed gluten exposure on the development of islet autoimmunity in children at increased risk for diabetes in Germany. Children younger than 3 months with at least one first-degree relative with T1D and one of three specific T1D-associated HLA genotypes (DRB1*03-DQA1*05:01-DQB1*0201/DRB1*04-DQA1*03:01-DQB1*03:02; DRB1*04-DQA1*03:01-DQB1*03:02/DRB1*04-DQA1*03:01-DQB1*03:02 or DRB1*03-QA1*05:01DQB1*02:01/DRB1*03-DQA1*05:01DQB1*02:01) were recruited between 2,000 and 2,006 (participation rate: 88.8 %) and randomized to exposure to dietary gluten from age 6 months or from age 12 months. After inclusion, children were followed in three monthly intervals until the age of 3 years and yearly thereafter for efficacy (persistent islet autoantibodies) and safety assessment, including intensive monitoring with three monthly sample collection of venous blood, urine and stool. PBMCs were isolated from venous blood samples taken at each visit and stored at −80 °C in TRIZOL.

The T1D PBMCs were collected as part of the Genetic Resource Investigating Diabetes (GRID)cohort collection ( http://www.childhood-diabetes.org.uk/grid.shtml) by our laboratory and others. Blood samples were collected in morning or afternoon hospital clinics, across more than 150 centres in the United Kingdom. Blood was collected into ACD vacutainers and PBMCs isolated separated using either Sigma Accuspin or Histopaque according to the manufacturer's recommendations. PBMCs were cryopreserved in the presence of DMSO and stored at −80 °C until use. For RNA isolation, PBMC samples were thawed, washed with X-Vivo 15 (Lonza) and added to Trizol reagent. RNA was isolated and gene expression data were generated in the same way as the BABYDIET cohort, described in the following section.

All BABYDIET and T1D PBMC gene expression data are deposited with ArrayExpress (accession number: E-MTAB-1724).

Gene expression data for the multi-centre asthma cohort is publically available. The cohort was collected and processed as described by Bjornsdottir *et al.*^34^, and the raw and normalized data are deposited with ArrayExpress ( http://www.ebi.ac.uk/arrayexpress/, E-GEOD-19301). This gene expression data set was generated on Affymetrix HG-U133A GeneChip Array, which we found to have 22,283 probesets (which map to 10,457 unique ENSEMBL gene identifiers). The inclusion of patients in the initial collection of the study was dependent on participants being free from active infection, major intercurrent illness, allergen immunotherapy, pregnancy and lactation^34^.

The available processed data as well as the R ExpressionSet file were downloaded from ArrayExpress. Information regarding the disease phase of the samples, their country of origin and the date of bleeding was used in our analyses. Only asthma patients defined as being in a quiet disease phase were included in our analyses. Precise age at bleed for each donor was also not available in this data set, although individuals between 18 and 83 years of age were present in the cohort. The mean age of the asthma patients was 45.08 years^34^.

As information regarding sample gender in this data set was not available, we defined gender based on Y-expressed genes in PBMCs (*DDX3Y*, *KDM5D*, *USP9Y*, *and RPS4Y1*). The first principal component of the expression of the listed genes was calculated for each individual in the study. Patients with component values smaller than zero were classified as female and patients with component values greater than zero were classified as male.

The asthma PBMC data set was divided into four groups according to country of sample collection; United States, Australia, United Kingdom/Ireland and Iceland.

The subcutaneous adipose tissue gene expression data were collected by the MuTHER consortium^53^ and is publically available (Array Express E-TABM-1140). The adipose tissue data set includes 825 female twins, among them 80 singletons, 448 dizygotic and 297 monozygotic individuals. Gene expression data for 48,638 probesets (mapping to 24,332 unique Entrez genes) were downloaded.

Sample numbers included in our analyses of each cohort, and their monthly distributions, are shown in Supplementary Table 1.

### Gene expression analysis in BABYDIET and T1D PBMCs

Only gene expression data for the BABYDIET and T1D PBMC cohorts were generated in our laboratory. In brief, single-stranded cDNA was synthesized from 200 ng total RNA using the Ambion whole-transcript expression kit (Ambion) according to the manufacturer's recommendations. A total of 3.44 μg cDNA was fragmented and labelled using the GeneChip terminal labelling and hybridization kit and hybridized to 96-sample Titan Affymetrix Human Gene 1.1 ST arrays, which provide comprehensive whole-transcriptome coverage. After quality control, we measured the expression of 33,297 probesets, which map to 22,822 unique ENSEMBL gene identifiers.

BABYDIET, T1D and adipose gene expression data were summarized by exon-level probesets and normalized using variance stabilizing normalization: post quality control 454 BABYDIET^35^, 236 T1D and 825 adipose samples were used for analysing gene expression.

The gene expression data of the asthmatic patients were log2 transformed before any analysis.

### Climatic data for modelling seasonal gene expression

Historical raw data for the mean daily temperature, as well as the total daily hours of sunlight in Munich (Germany), were obtained from the Integrated Climate Data Centre at the University of Hamburg ( http://icdc.zmaw.de/dwd_station.html?&L=1).

For the analysis of the T1D PBMC data that came from all around United Kingdom we downloaded the maximum and minimum temperature data from seven stations across United Kingdom (Armagh, Camborne, Eskdalemuir, Lerwick, Stornoway airport and Valley) from the National Climatic Data Centre, USA ( http://www.ncdc.noaa.gov/cdo-web/search) and averaged readings across all stations.

For the analysis of the asthma cohort (ArrayExpress: E-GEOD-19301), the daily maximum and minimum temperature for relevant cities/regions in the United Kingdom (Central England UK station at Birmingham), United States (New Jersey, Seattle, Atlanta, New Haven), Iceland (Reykjavik), Ireland (Dublin) and Australia (Melbourne, Perth, Adelaide) were obtained from the National Climatic Data Centre, USA and The Digital Technology Group. The average temperature values were computed and used in subsequent analyses.

### Self-reported infections in BABYDIET cohort

At each visit, parents of BABYDIET children completed a detailed questionnaire on their children's history of infections, fever and medication. Specifically, they were asked about fever, infectious symptoms (such as diarrhoea, vomiting, constipation and allergies) and the name of administered pharmaceutical agents or their active ingredient with starting date and duration of infections and medication. Infectious disease was defined as an acute event according to the ICD710 Code or by a symptom indicating an infectious genesis. Infectious events were assigned to a specific time interval by their date of onset, and infectious events that could be matched to microarray samples were included for analysis, as described^35^. Other disease events such as allergies or accidents were not considered as infectious diseases.

### Soluble IL-6 receptor ELISA

Circulating sIL-6R concentrations were measured in BABYDIET and BABYDIAB serum samples using a highly sensitive non-isotopic time-resolved fluorescence ELISA assay based on the dissociation-enhanced lanthanide fluorescent immunoassay technology (DELFIA; PerkinElmer), as described^45^. Test samples were diluted 1:20 in PBS+10% FBS and measured in duplicate on 384-well MaxiSorp microtiter plates (Nunc), coated with 1 μg ml^−1^ monoclonal anti-human IL-6R antibody (clone 17506; RD Systems). Detection was performed using a biotinylated mouse anti-CD126 monoclonal antibody (clone M182, BD Biosciences) diluted to a final concentration of 100 ng ml^−1^ in PBS+10% FBS and a Europium-Streptavidin detection solution (PerkinElmer), diluted in PBS+0.05% tween, 1% BSA, 7 μg ml^−1^ DTPA to a final concentration of 0.05 μg ml^−1^. Quantification of test samples was obtained by fitting the readings to a human recombinant IL-6Rα (RD systems) serial dilution standard curve plated in quadruplicate on each plate. Data for 782 unique individuals existed from 722 families.

### Cambridge BioResource full blood count data (UK cohort)

Full blood count data were obtained from the Cambridge BioResource. BioResource volunteers are subjected to a full blood count on the day of blood sample collection using Beckman Coulter LH700, Beckman Coulter DXH800 5 part diff analyser or a Sysmex 5 part diff analyser. The available months of bleed were from February to November (no FBC data was available for December) and took the numeric values 2 to 11, respectively. Responses measured included counts for basophils, eosinophils, lymphocytes, monocytes, neutrophils, platelets, erythrocytes and total white blood cells. HCT (haematocrit), HGB (haemoglobin concentration), MCH (mean corpuscular haemoglobin) and MCV (mean corpuscular volume) were also analysed.

### Full blood count data from The Gambia

The Gambian cohort was collected as part of the Keneba Biobank ( http://www.ing.mrc.ac.uk/research_areas/the_keneba_biobank.aspx). All participants were recruited between 2012 and 2014 in the West Kiang district and within the catchment area of the MRC International Nutrition Group's field station at MRC Keneba. Supplementary Figure 7 gives summary statistics for the cohort. Written informed consent was obtained from all participants and all procedures were approved by the joint Gambian Government/MRC Ethics Committee. FBCs were available from 4,200 healthy individuals (at the time of sample collection; 44.07% male) using a Medonic M-series analyser, which measures the numbers of white blood cells, lymphocytes, granulocytes, monocytes, platelets and RBCs. Furthermore, it also analyses the mean platelet volume, RBC haemoglobin concentration, the haematocrit, MCV and MCH.

### C-reactive protein

The level of CRP in the peripheral circulation was measured in 3,412 donors (two samples per donor) collected as part of the ASCOT study^68^. Treatment with Atorvastatin did not remove the seasonal variation in this parameter. Age and sex were included as covariates, while a random intercept was added for the individual identifiers.

### Statistical analysis of the data sets

Cosinor models with a period of 1 year were fitted to test the effect of season on gene expression. The general formula of the fitted model is given by:


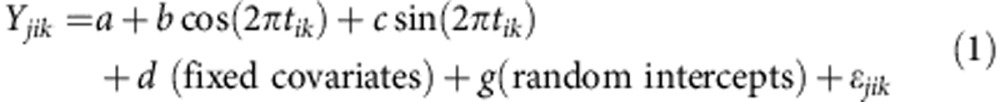


where *Y*_*jik*_ represents the log2 expression of gene *j* for individual *i* recorded at time *t*_*ik*_, with *t*_*ik*_ computed as the calendar day of the date of bleed divided by the total number of days within the equivalent year.

The fixed covariates and random intercepts terms were data-set-specific. For the analysis of the BABYDIET and T1D data sets we added age at bleed and gender as fixed effects covariates, whereas only gender was added as a covariate in the analysis of the asthma PBMC microarray dataset (age was not available). The identity of each subject of the BABYDIET and of the asthma data sets were modelled as a random intercept in the corresponding models. For the adipose tissue data set we modelled age at bleed as a fixed covariate and added family identity and an indicator whether the twin was monozygotic or dyzogitic as random intercepts. Gender and age at bleed were treated as fixed effects covariates in the analysis of the soluble IL-6 receptor data, and family identity was included as a random intercept. As only the month of bleed was available in the Cambridge BioResource FBC data, we adjusted the cosinor model to depend on month instead of day; no other covariates were available and random intercepts were not required, as no individual was observed more than once. For the last two data sets, the response variable (*Y*) corresponds to IL-6R and to the tested FBC responses listed in the description of the data set. For analysis of CRP, age, sex and an age*sex interaction were included as fixed covariates, CRP was log transformed to remove right skew, and a random intercept was used to adjust for within individual repeated measures.

To examine whether the effect of season was significant we compared the fitted model in equation (1) with a model that did not include the effect of season. This alternative model is expressed by


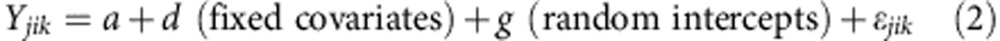


The *P*-value for season was determined by comparing the two models for each gene using an analysis of variance test. Seasonal genes were classified as those with *P* values less than the data-set-specific Bonferroni correction threshold alpha=0.05. For the BABYDIET and T1D data sets, we defined as seasonal the genes with *P* values less than the corresponding Bonferroni correction *P* value and with mean log2 expression greater than or equal to, 6.

The relative estimated log2 expression of each seasonal gene for each data set was computed as


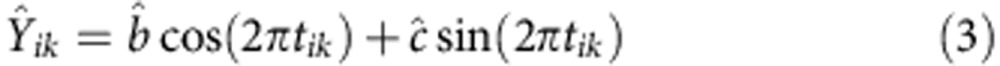


where 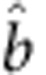 and 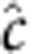 are the least squares estimates of *b* and *c* of the model in equation (1), respectively.

Furthermore, we tested whether temperature or sunlight hours could predict gene expression of the PBMC data sets. Temperature and sunlight were defined, respectively, as the average temperature and number of sunlight hours over the week preceding the date of bleed for each individual. For example the temperature model is given by


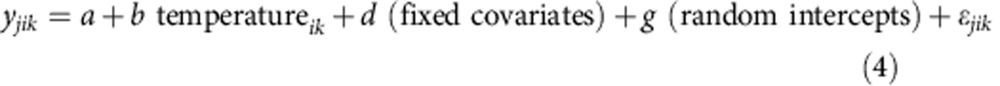


The three alternative models for the seasonal cosinor function, sunlight and temperature, each including only one of these predictors were fitted to log2 expression level for seasonal genes, as identified in each data set.

### Definition of winter and summer seasonal genes in BABYDIET

Seasonal genes were classified as winter genes if the relative estimated log2 expression values of the genes were positive for all days of January, February and December and negative for all days of June, July and August. In contrast, summer seasonal genes were defined as those with positive relative estimated log2 expression for all days of June, July and August and negative for all days of January, February and December. The fold change for each summer and winter gene was computed as two raised to the power of the absolute difference of the estimated log2 expression between 15 January and 15 July (days 15 and 196 of a 365-day calendar year).

### Network and functional analysis of the seasonal genes identified in BABYDIET

A weighted co-expression gene network of the seasonal genes identified in BABYDIET was constructed using the R package WGCNA^69^. For the construction of the network, individuals who sero-converted to T1D autoantibodies at any stage during the BABYDIET study were not included. A scale-free topology network was created based on the seasonal genes, where the correlation of their log2 gene expression was used as a measure of co-expression. Modules of highly correlated genes were detected through hierarchical clustering. Some genes were not correlated with other seasonal genes. The biological function of each module was examined through an over-representation pathway analysis carried out using the WEB-based GEne SeT AnaLysis Toolkit (WebGestalt, http://bioinfo.vanderbilt.edu/webgestalt/)^70^. The gene members of each module were uploaded to WebGestalt and tested for over-representation within KEGG pathways. Pathways with less than three genes within our gene lists were excluded. The Hypergeometric test was applied, and the *P* values of the test were corrected for multiple testing using the Benjamini–Hochberg method.

### Analysis of the self-reported infections data of BABYDIET cohort

The BABYDIET samples were divided into two categories, one that included all samples with no self-reported infections (57 samples) and one with all the samples with at least one reported infection (152 samples). A principal component analysis (PCA) was performed, and the first principal component from the analysis was used to summarize the gene expression of BABYDIET seasonal genes. Similarly, the gene expression of the genes within the black module (detected using network analysis of the seasonal genes identified in BABYDIET) was also summarized as the first component of a second PCA. The effect of infection on either of the two components was tested using analysis of variance. The black module was chosen as it contained genes associated with the response to *Staphylococcus* infection.

### Identification of common seasonal genes

We wanted to explore whether any of the seasonal genes identified in the PBMC cohorts were shared between the five data sets (excluding Iceland). We compared the Bayesian information criterion (BIC) of the cosinor model (1) with the BIC of the model excluding the seasonality effect (2) for each of the genes from the two in-house data sets (BABYDIET and T1D) that had a *P* value <0.05/33297 in at least one of the two data sets. The common seasonal genes of the two in-house datasets were defined as genes whose BIC was smaller for (1) than (2) within each data set. We repeated the aforementioned steps to identify common seasonal genes in the asthma cohort. The intersection of the two lists from the five data sets were defined as common seasonal genes.

We further computed a combined *P* value for the association of each common seasonal gene by combining the *P* values of the five data sets using Fisher's product *P* value method.

Common seasonal genes between the adipose tissue data set and the BABYDIET data set were defined as the genes that were found seasonal for both data sets.

### Seasonal analysis of The Gambian full blood count data

Given the different seasonal climates present in West Africa compared to Europe, FBC parameters from The Gambia cohort were assessed through linear models that included sex, age (modelled through splines) and with seasonality modelled using three Fourier terms using STATA12.1. The significance of season was assessed using an F-test.

### Note added in proof

Adaptive oscillations at balanced polymorphisms in *Drosophila* in response to acute and persistent changes in climate were reported while this work was under consideration (Bergland, A.O., Behrman, E.L., O'Brien, K.R., Schmidt P.S. & Petrov D.A. *Plos Genet.* 10(11):e1004775 (2014)). Furthermore, seasonally-variable associations of three genes involved in glucose metabolism and circadian clock regulation, *CRY1* (cryoptochrome 1), *CRY2* (cryoptochrome 1) and *MTNR1B* (melatonin receptor 1B) have recently been reported in humans. (Renström, F., Koivula, R.W., Varga, T.V., Hallmans, G., Mulder, H., Florez, J.C., Hu, F.B. & Franks, P.W. *Diabetologia* 10.1007/s00125-015-3533-8 (2015)).

## Author contributions

X.C.D. had the idea for the study. X.C.D., M.E., C.W. and J.A.T. designed experiments and analysed the data. M.E., H.G., N.C. and O.S.B. performed statistical analyses. C.W. supervised the statistical analyses. X.C.D. wrote the manuscript with contributions from M.E., E.B., A.-G.Z., C.W. and J.A.T.; R.C.F., M.L.P. and D.J.S. performed wet lab or *in silico* experiments and assisted with data processing. A.J.F., B.J.H. and A.M.P. were involved in the generation and analysis of blood count data from The Gambia. A.-G.Z. is the PI of the BABYDIET study and takes responsibility of the integrity of the data; A.-G.Z. and E.B. designed the BABYDIET protocol, provided samples and clinical data.

## Additional information

**Accession codes**: Mircoarray data have been deposited in ArrayExpress under accession code E-MTAB-1724.

**How to cite this article:** Dopico, X. C. *et al.* Widespread seasonal gene expression reveals annual differences in human immunity and physiology. *Nat. Commun.* 6:7000 doi: 10.1038/ncomms8000 (2015).

## Supplementary Material

Supplementary Information Supplementary Figures 1-9.

Supplementary Dataset Supplementary dataset tables.
