# Supplementary material for: Widespread seasonal gene expression reveals annual differences in human immunity and physiology
Source: Nat Commun. 2015 May 12;6:7000. doi: 10.1038/ncomms8000 (PMC4432600; doi:10.1038/ncomms8000)
Supplement: Supplementary Information — Supplementary Figures 1-9. [file ncomms8000-s1.pdf]

Supplementary Figure 1

Fold changes in seasonal gene expression (BABYDIET dataset)

	Minimum	1st Qu.	Median	Mean	3rd Qu.	Maximum
**Summer genes**	1.0481	1.1626	1.2185	1.2572	1.3048	5.6423
**Winter genes**	1.0618	1.1741	1.2516	1.3150	1.3806	3.4011

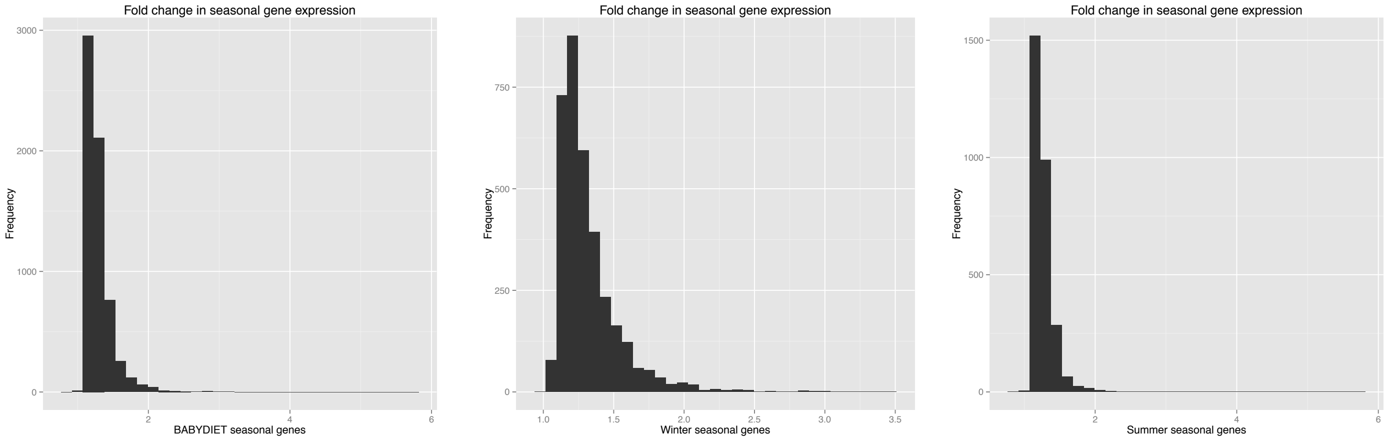

<u>Supplementary Figure 1 – Fold change in seasonal gene expression</u>

The minimum, 1st quartile, median, mean, 3rd quartile and maximum cosine fold changes in gene expression, of all genes defined either as winter or summer in the BABYDIET dataset are shown. Fold change in gene expression is defined as two raised to the absolute difference of the estimated log2 expression between the 15th January and the 15th July (the mid-points of summer and winter). Histograms of the frequency of fold-changes in expression amongst seasonal genes.

Supplementary Figure 2

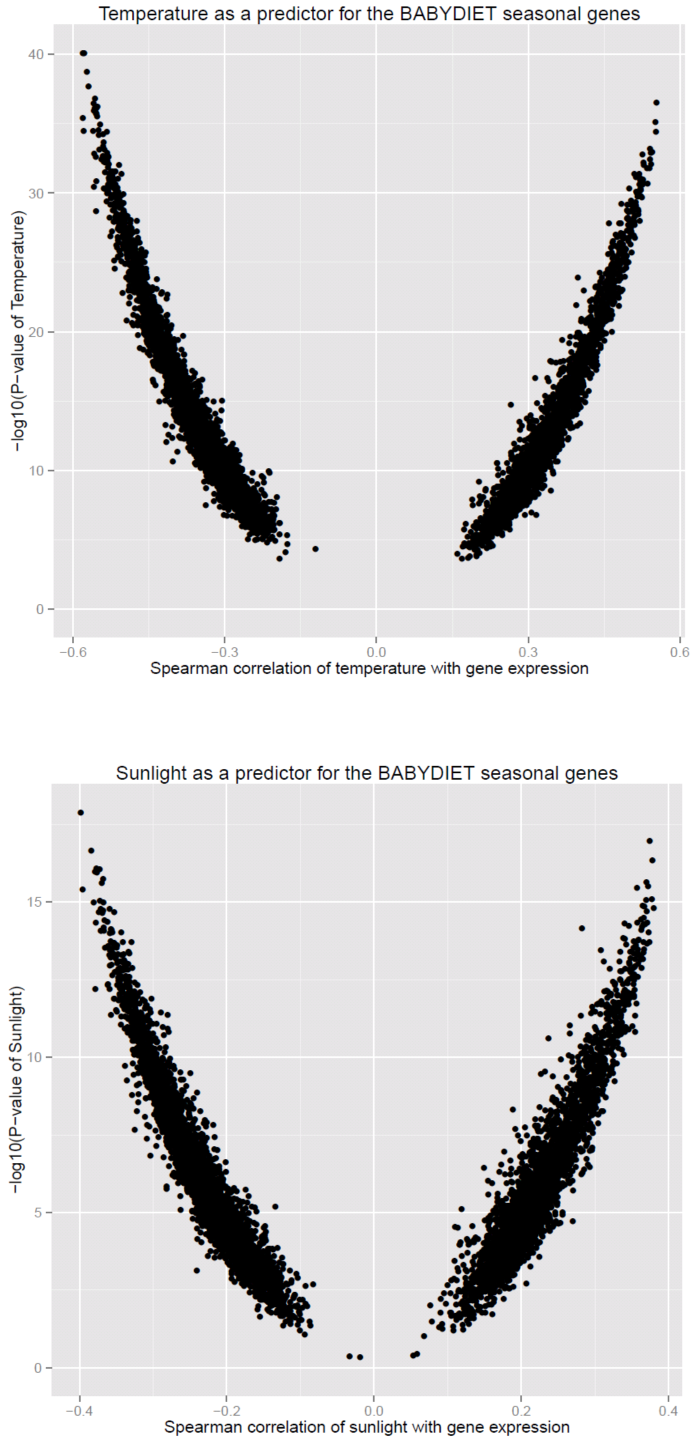

<u>Supplementary Figure 2 – Temperature and sunlight as predictors of seasonality</u>

Volcano plots of the *P*-values of association of daily mean temperature and daily mean sunlight hours against their corresponding Spearman correlations with gene expression: for the BABYDIET seasonal genes, suggesting a strong association with both predictors.

Supplementary Figure 3

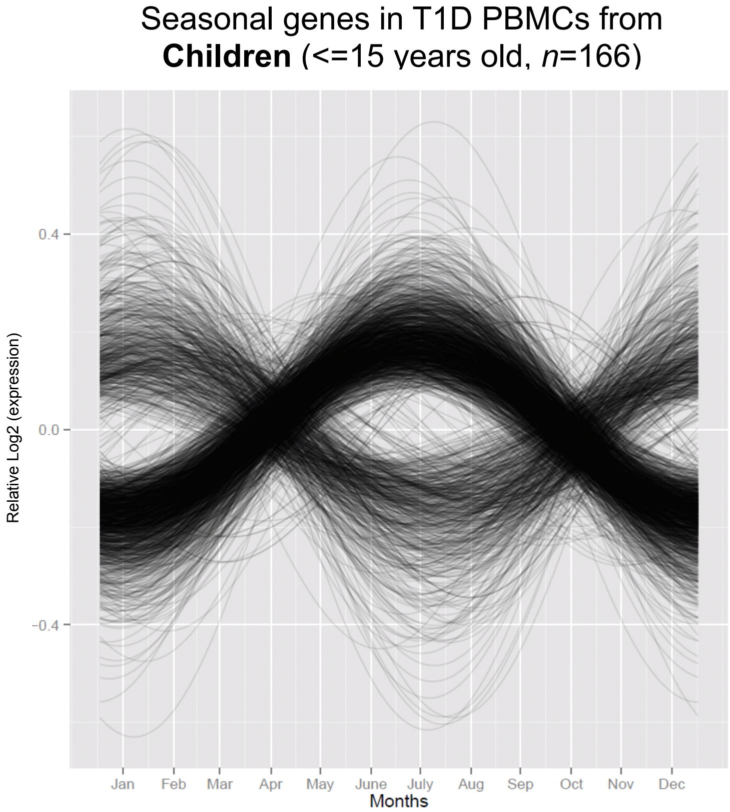

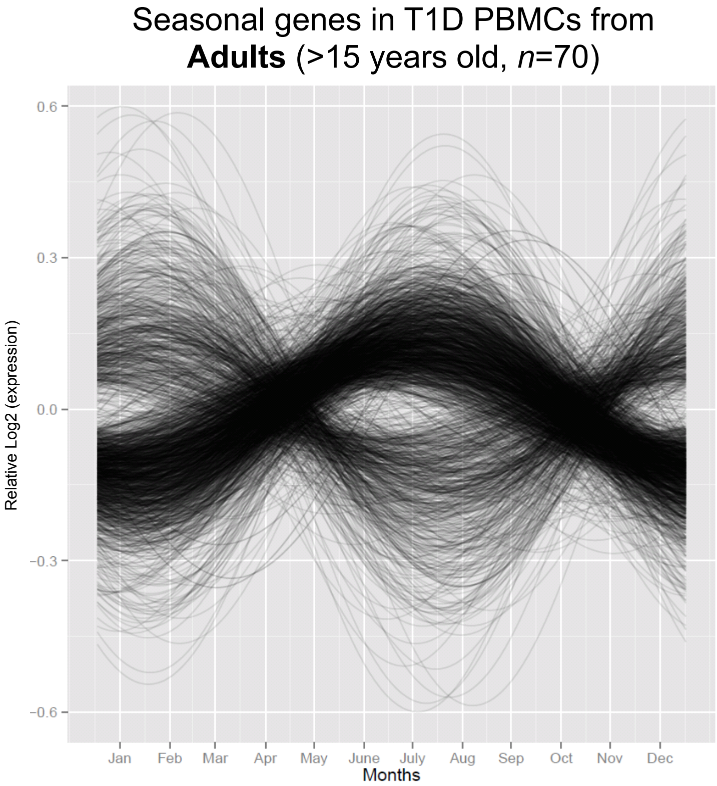

Supplementary Figure 3 – Seasonal gene expression replication in children and adult T1D PBMCs

We were able to replicate our observation of seasonal gene expression in a unique collection of 236 T1D PBMC samples from children and adults, in which 1,697 genes were found to be seasonal. The estimated log2 expression of these seasonal genes was used to display seasonal gene expression in either children (<=15 years old) or adults (>15 years old) from this dataset. We observed seasonal gene expression in adults and children. Importantly, the seasonal genes in the adults differed from those in the children.

Supplementary Figure 4

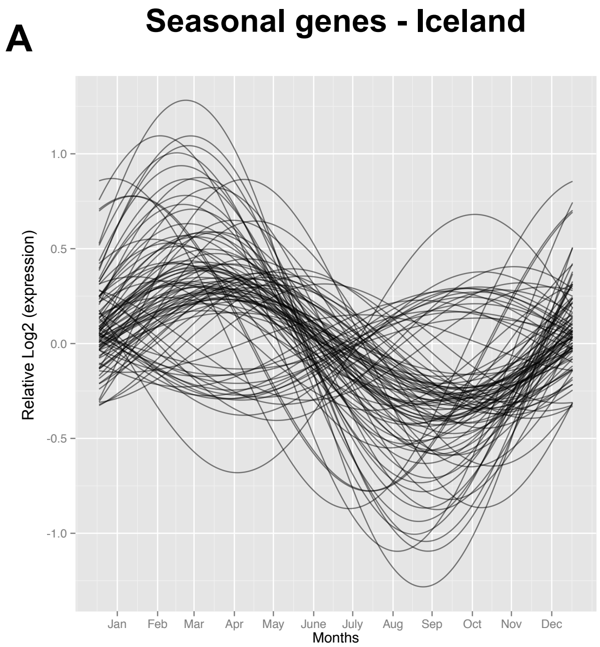

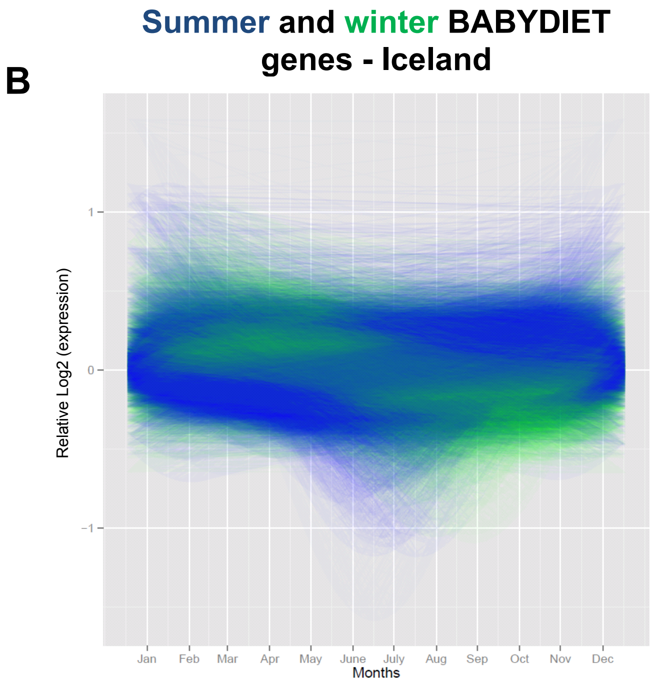

<u>Supplementary Figure 4 – Seasonal gene expression in asthmatic patient PBMCs</u>

(A) Seasonal genes in the Iceland cohort are displayed (68 genes). The seasonal pattern of these genes was distinct from those of all other cohorts, with the absence of the typical difference in expression lying between summer and winter months. (B) BABYDIET summer and winter seasonal genes also did not have the same clear seasonal preference in the Icelandic samples as in all other cohorts.

Supplementary Figure 5

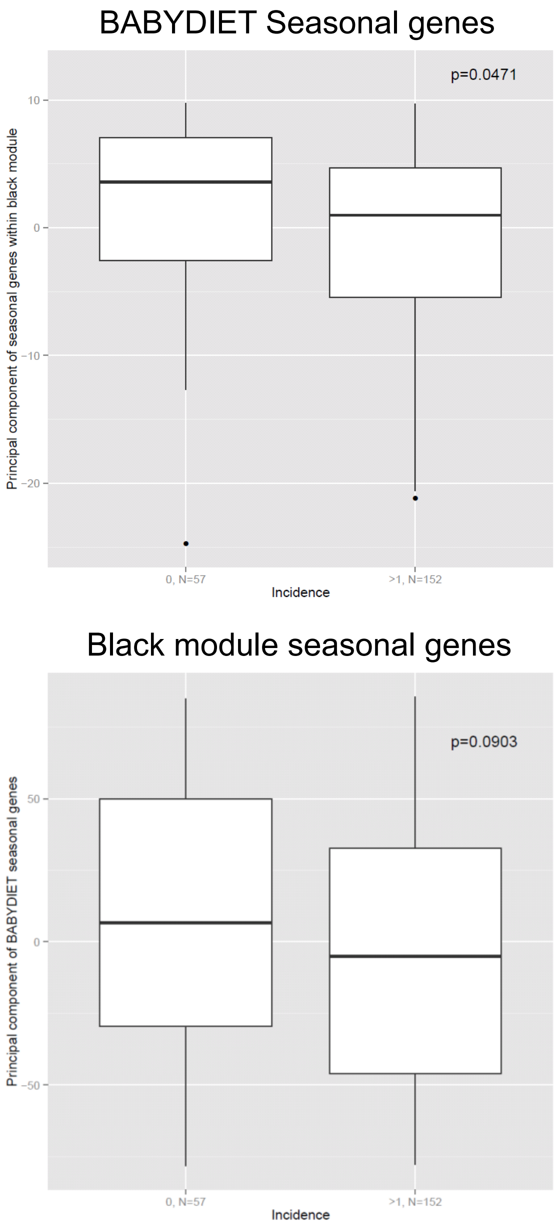

<u>Supplementary Figure 5 – Self-reported infections do not fully explain seasonality</u>

The first principal component of the expression of the total seasonal BABYDIET genes, and of the genes contained within the black co-regulated module, were tested with association with infection. Infection data were collected in the BABYDIET dataset as self-reported infections, and samples collected within 1.5 months of a self-reported infection were compared to samples with no self-reported infections. The black module contains genes involved inflammatory responses.

Supplementary Figure 6

A Common seasonal genes in each cohort

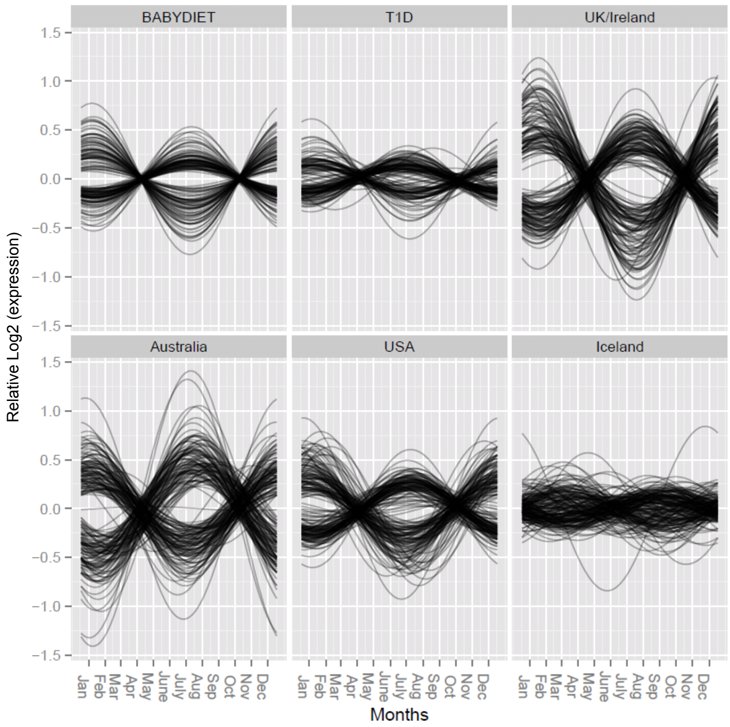

B *ARNTL* expression in each cohort

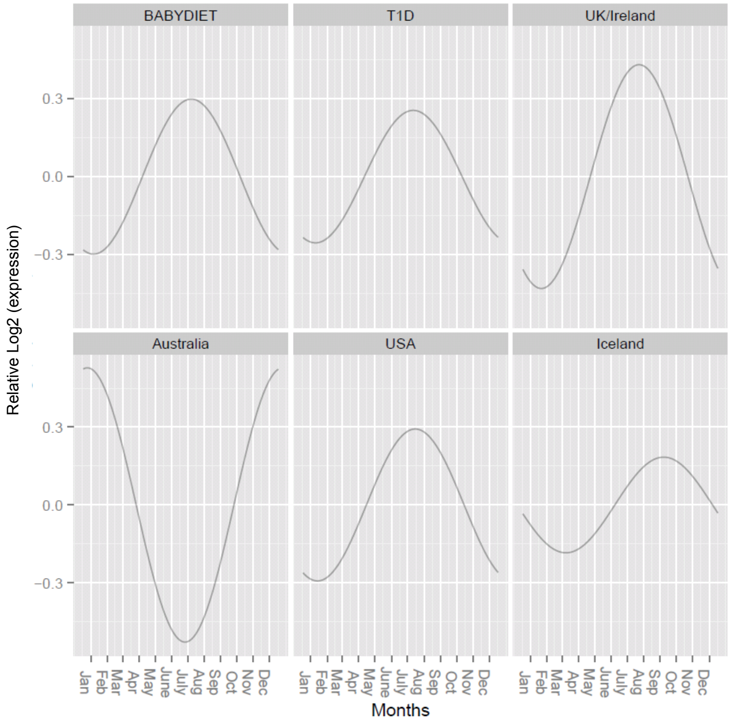

Supplementary Figure 6 - Common seasonal genes in the peripheral immune system
147 genes were found to be seasonal in PBMCs in the BABYDIET, T1D, Australian, USA and UK/Ireland cohorts. (A) These genes showed similar seasonal patterns in all datasets, except for Iceland. (B) *ARNTL* (combined P=6.73x10^{-57}) was found amongst the common seasonal genes, and its expression was increased in the summer months in all datasets, except in Iceland where *ARNTL* had a more complex seasonal expression profile.

Supplementary Figure 7

A

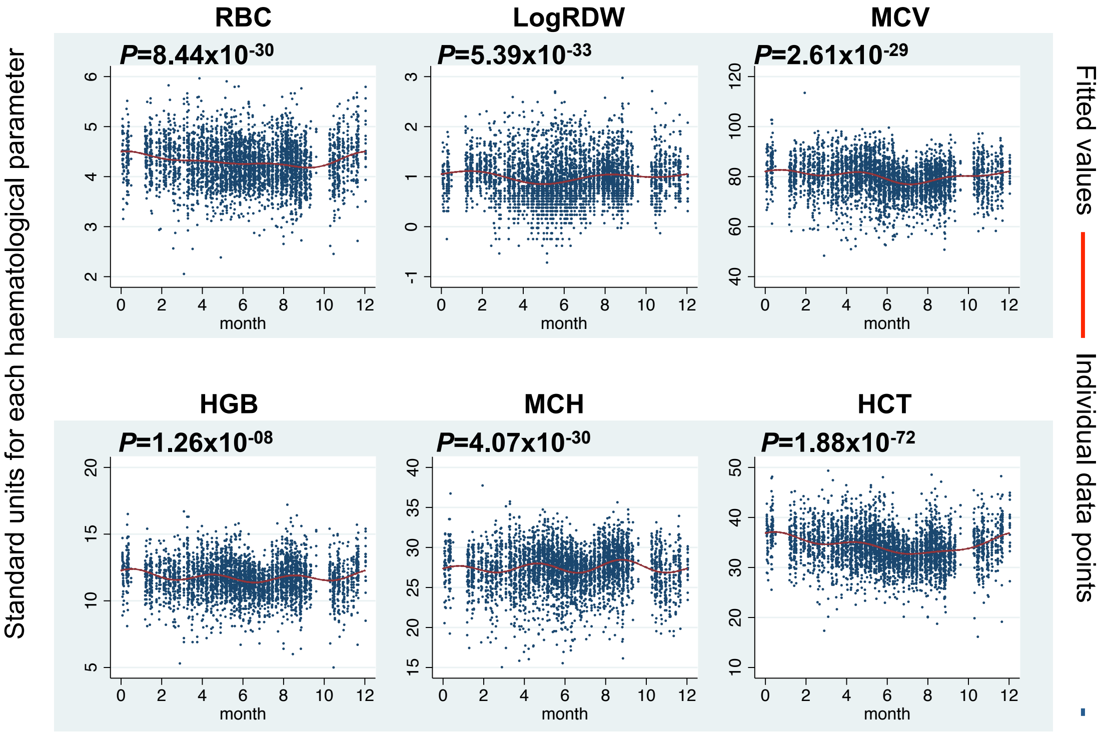

B

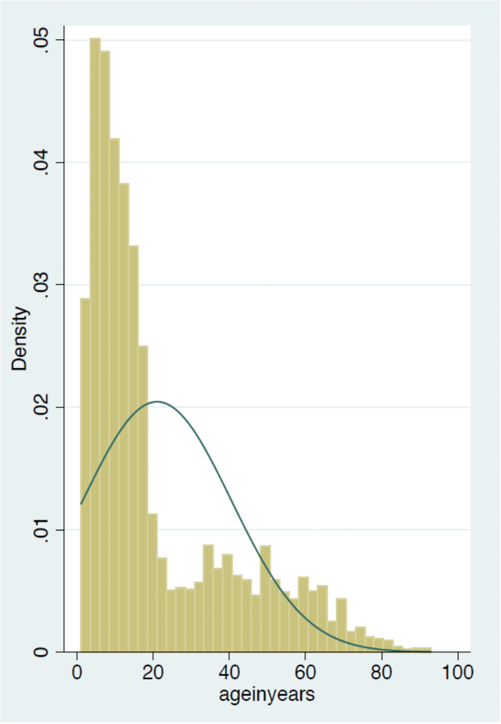

C

Variable	Observations	Mean	SD	Min	Max
Age [years]	4200	21.02	19.21	1.01	92.96
RBC [n/l]	4200	4.29	0.44	2.08	5.99
logRDW [%]	4200	15.00	1.58	12.50	32.10
MCV [fl]	4197	79.83	6.95	48.90	114.00
HGB [g/dl]	4191	11.75	1.30	5.10	17.30
MCH [pg]	4189	27.53	2.68	15.20	37.90
MCHC [g/dl]	4189	34.47	1.43	28.50	40.10
LYM [n/l]	4157	2.57	0.99	0.60	10.80
GRAN [n/l]	4155	3.25	1.34	0.80	13.90
MONO [n/l]	4158	0.51	0.16	0.20	1.60
HCT [%]	4197	34.14	3.81	16.40	49.60
PLT [n/l]	4197	333.69	101.53	11.00	968.00

Supplementary Figure 7 – The Gambia full blood count data

(A) Red blood cell parameters were found to be seasonal in addition to the white blood cell parameters reported in the main text and Fig. 4C. (B) Age (in years) distribution of the Gambian cohort. (C) A demography table of the Gambian samples.

Supplementary Figure 8

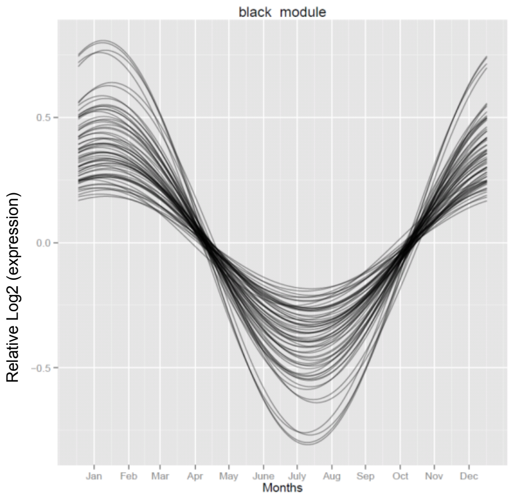

**Black module**<br>**Increased WINTER expression**		
Pathway	Genes	P-Value (adj.)
Staphylococcus aureus infection	*FCGR3A, FPR1, FPR2*	0.0002
Cytokine-cytokine receptor interaction	*TNFRSF10C, CXCR1, CXCR2, CSF3R*	0.0006
Metabolic pathways	*MGAM, CYP4F3, LPCAT2, ALPL, HAL*	0.0167

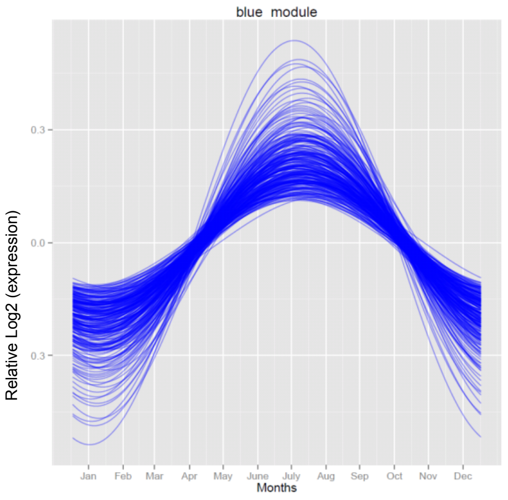

**Blue module**<br>**Increased SUMMER expression**		
Pathway	Genes	P-Value (adj.)
Ubiquitin mediated proteolysis	CUL5, UBE3A, SMURF1, HERC1, BIRC2, UBR5, CUL3	0.0002
RNA Transport	*RPP30, NUP155, NUP153, TGS1, SUMO4, NUP133, NUP98*	0.0002
Circadian rhythm - mammal	*CRY1, RORA, ARNTL*	0.0011
Splicesome	*AQR, TCERG1, SRSF4, CRNKL1, PRPF40A*	0.0030
Ribosome biogenesis - eukaryotes	*RPP30, HEATR1, DKC1, GNL3*	0.0034
Proteasome	*PSMD11, PSMD12, PSME4*	0.0054
RIG-I-like receptor signalling pathway	*RIPK1, MAPK8, TRAF3*	0.0124
Hepatitis C	*RIPK1, MAPK8, TRAF3, BRAF*	0.0124
Aminoacyl-tRNA biosynthesis	*RARS, SPESECS, RARS2*	0.0124
RNA degradation	*CNOT2, CNOT6L, DHX36*	0.0124

Supplementary Figure 8

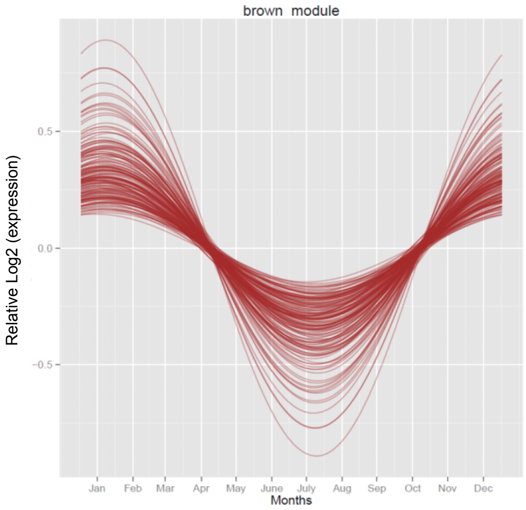

Brown module
Increased WINTER expression

Pathway	Genes	P-Value (adj.)
Osteoclast differentiation	*LILRB2, NCF4, LILRA1, FCGR2A, LILRB1, LILRA6, LILRA2, NCF2, SYK, CSF1R, CYBB*	1.75e-11
Metabolic pathways	*AMPD2, TBXAS1, CDA, AGPAT1, SPTLC2, AKR1A1, ALOX5, GNS, GBGT1, ACSS2, G6PD, IMPDH1, COMT, GLB1, TALDO1, HK3, NADK, TPI1,*	7.06e-07
Leishmaniasis	*JAK2, NCF4, CR1, PTPN6, NCF2, FCGR2A*	1.21e-06
FC gamma R-mediated processes	*GSN, VASP, HCK, SYK, PRKCD, FCGR2A*	4.45e-06
Phagosome	*SCARB1, NCF4, CD36, NCF2, FCGR2A, CYBB*	5.97e-05
Axon guidance	*CDK5, SRGAP2, SEMA4A, DYPSL2, FES*	0.0002
Glycolysis/ gluconeogenesis	*HK3, ACSS2, TPI1, AKR1A1*	0.0002
Fat digestion/absorption	*SCARB1, CD36, AGPAT1*	0.0011
Chemokine signalling pathway	*JAK2, FGR, HCK, ADRBK2, PRKCD*	0.0011
Glutathione metabolism	*GSR, G6PD, MGST1*	0.0012

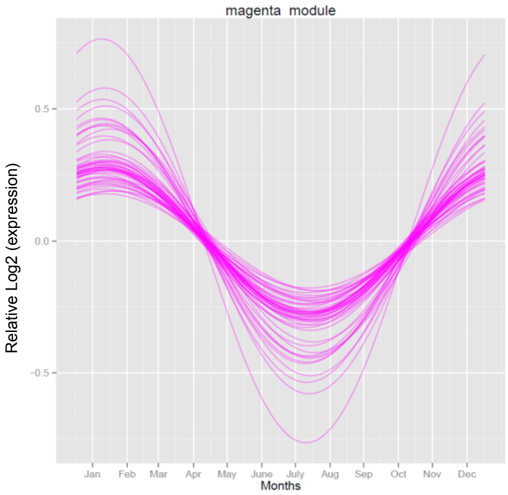

Magenta module
Increased WINTER expression

Pathway	Genes	P-Value (adj.)
Haematopoietic cell lineage	*FCER2, CD19, CD22, MS4A1*	9.40E-06
B cell receptor signalling pathway	*CD19, CD22, PLCG2*	0.0001
Phosphatidylinositol signalling system	*PLCG2, ITPR1, PIP5K1B*	0.0001
Cell adhesion molecules (CAMs)	*HLA-DOA, HLA-DOB, CD22*	0.0004
Calcium signalling pathway	*P2RX5, PLCG2, ITPR1*	0.0007

Supplementary Figure 8

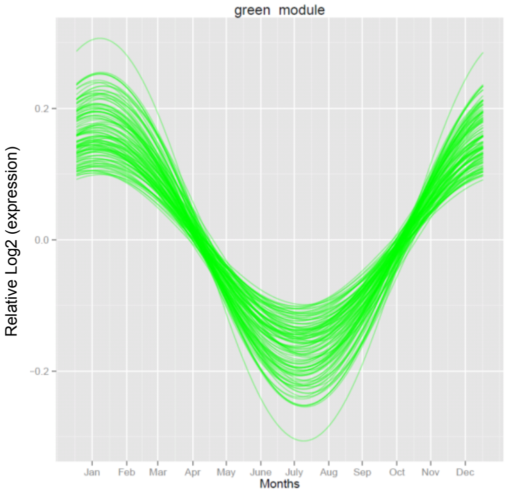

Green module
Increased WINTER expression

Pathway	Genes	P-Value (adj.)
Metabolic pathways	*ATP5G1, POLR3D, TSTA3, UCKL1, MRI1, POLR2E, ACLY, APRT, GPAA1, UQCRC1, DGAT1, PIP5K1C*	8.40e-05
Regulation of actin cytoskeleton	*PIP4K2B, ITGB7, CYFIP2, MAPK3, FGD3, PIP5K1C*	8.40e-05
Insulin signalling pathway	*EXOC7, TSC2, PRKCZ, MAPK3*	0.0015
Wnt signalling pathway	*NFATC3, PPP2R5D, CTBP1, CCND3*	0.0015
B cell receptor signalling pathway	*NFATC3, CARD11, MAPK3*	0.0021
Huntington's disease	*ATP5G1, UQCRC1, POLR2E, DCTN1*	0.0022
Focal adhesion	*ITGB7, CCND3, MAPK3, PIP5K1C*	0.0026
Pyrimidine metabolism	*POLR3D, UCKL1, POLR2E*	0.0030
T cell receptor signalling pathway	*NFATC3, CARD11, MAPK3*	0.0033
MAPK signalling pathway	*CDC25B, MAP4K1, MAP3K11, MAPK3*	0.0053

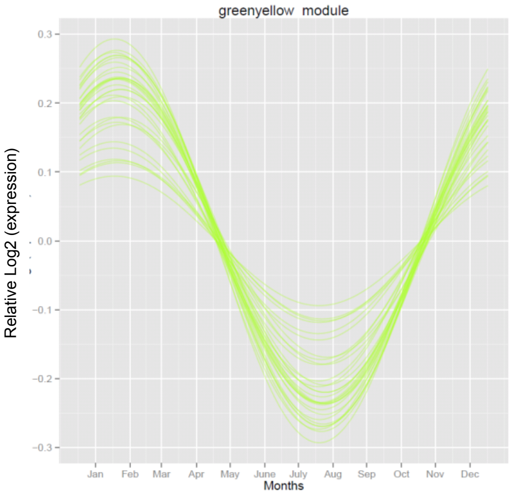

Green/Yellow module
Increased WINTER expression

Co-regulated probes in the green/yellow module did not map to currently annotated gene products

Supplementary Figure 8

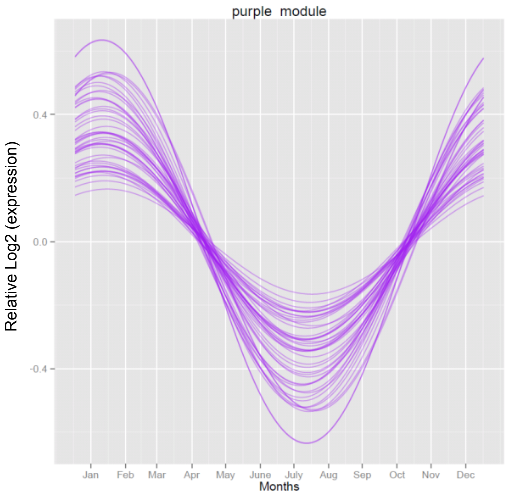

Purple module
Increased WINTER expression

Pathway	Genes	P-Value (adj.)
B cell receptor signalling pathway	*CR2, FCGR2B, CD72, BLNK2, CD79B, BTK,*	3.39e-10
Primary immunodeficiency	*BLNK, CIITA, BTK*	8.25e-06
Osteoclast differentiation	*FCGR2B, BLNK, BTK*	0.0003

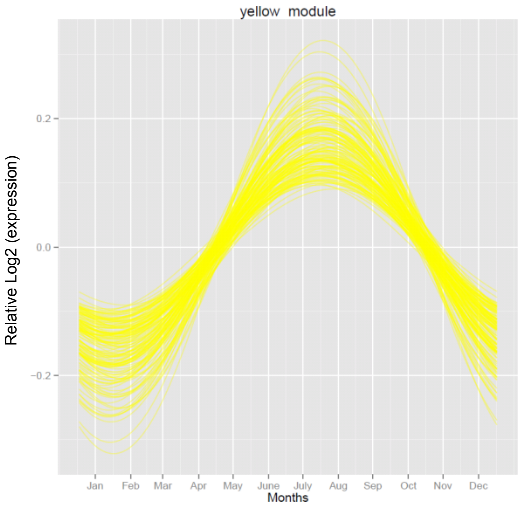

Yellow module
Increased SUMMER expression

Pathway	Genes	P-Value (adj.)
RNA transport	*RANBP2, EIF3J, RAE1, NUP54, DDX20, STRAP, NUPL1, PAIP1*	1.70e-07
RNA degradation	*DCP1A, HSPA9, DIS3, DDX6, CNOT8*	1.02e-05
Splicesome	*DHX8, SNW1, SYF2, SLU7*	0.0014
Acute myeloid leukaemia	*RPS6KB1, NFKB1, PIK3CA*	0.0014
Prostate cancer	*CTNNB1, NFKB1, PIK3CA*	0.0040
Osteoclast differentiation	*CYLD, NFKB1, PIK3CA*	0.0079
Neurotrophin signalling pathway	*FRS2, NFKB1, PIK3CA*	0.0079
Pathways in cancer	*CTNNB1, CUL2, NFKB1, PIK3CA*	0.0173

Supplementary Figure 8

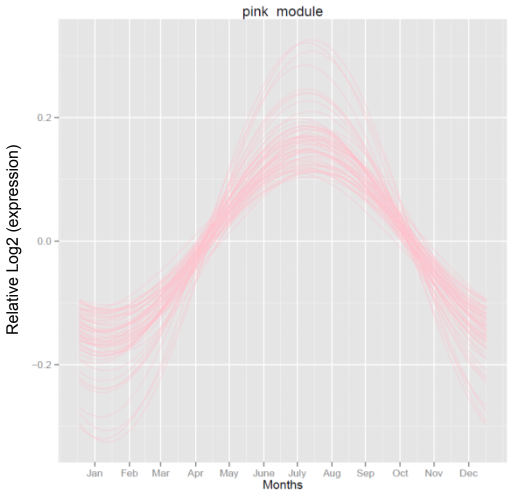

Pink module<br>Increased Summer expression		
Pathway	Genes	P-Value (adj.)
Co-regulated genes present in the green/yellow module did not map to known KEGG pathways under our analyses conditions		

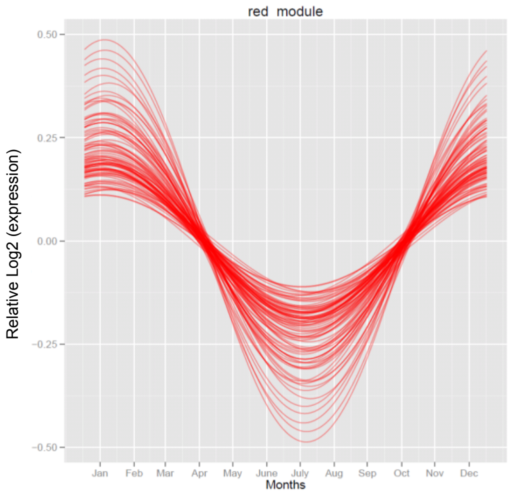

Red module<br>Increased WINTER expression		
Pathway	Genes	P-Value (adj.)
Lysosome	*TPP1, GAA, CTSS, SORT1, ARSB,*	2.96e-05
Leukocyte transendothelial migration	*ITGB2, GNAI2, MSN, ITGAM*	0.0002
Regulation of actin cytoskeleton	*ITGB2, MYH9, RRAS, MSN, ITGAM*	0.0002
Cell adhesion molecules (CAMs)	*ITGB2, CD4, VCAN, ITGAM*	0.0003
Hematopoietic cell lineage	*CD4, ITGAM, CD33*	0.0011
Amoebiasis	*ITGB2, PRKACA, ITGAM*	0.0016
MAPK signalling pathway	*ARRB1, RRAS, PRKACA, MAPKAPK3*	0.0022
Tight junction	*MYH9, RRAS, GNAI2*	0.0023
Phagosome	*ITGB2, CTSS, ITGAM*	0.0032
Metabolic pathways	*ITPK1, GAA, ALDH2, LTA4H, ARSB, BST1, TKT*	0.0045

Supplementary Figure 8

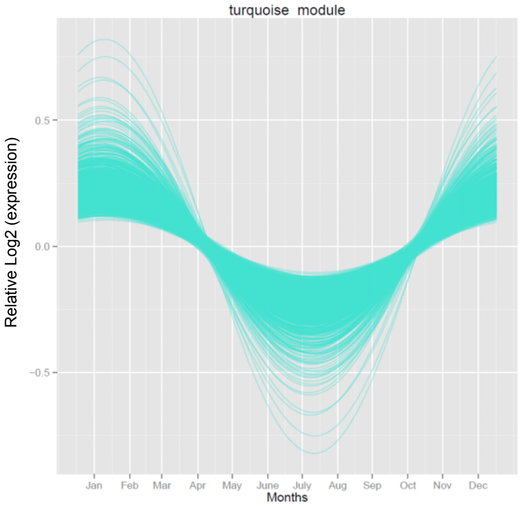

Turquoise module
Increased WINTER expression

Pathway	Genes	P-Value (adj.)
Metabolic pathways	*MPI, COX15, AK2, PIGN, PGM2, UGP2, DHCR24, UGDH, NDUFB5, ALG8, DPAGT1, AHCYL2, NDUFA2, INPP4A, ALDOC, DAD1, FUT8, SHMT2, HIBCH, DPYD, AHCYL1, ALDH18A1, GMDS, POLD4, PLCB2, ATP6V0E2, ALDH3A2, DNMT1, HSD17B12, MTHFD1, NT5E, PNPO, ACSL5, PDHA1, COQ5, AK5, PIGK, BCKDHB, POLA1, ECHS1, GALK2, OGDH, HSD17B4, IMPDH2, IDH1, GMPPA, PRIM1, GLCE, CAT, REV3L, ALAD, PRPS2, NNT, AGMAT, INPP4B, LPCAT4, ACADM, HMBS, ACSS1, ATP6V0E1, SCLY, HIBADH*	5.48e-18
Regulation of actin cytoskeleton	*ABI2, RAC2, ITGAL, WAS, SOS1, ARHGEF6, ARPC5, ITGA4, IQGAP1, PDGFD, PPP1CA, MAPK1, DIAPH1, PIP4K2A, ROCK2, IQGAP2,*	2.04e-06
MAPK signalling pathway	*PPP3CA, SOS1, RPS6KA1, TAOK1, HSPA8, TP53, NLK, DUSP22, RASA1, RAC2, PRKCB, MAP2K6, IKBKB, MAP3K12, HSPB1, MAPK1, PRKACB, TAOK3*	2.04e-06
Purine metabolism	*GMPR2, PDE6D, AK2, PGM2, AK5, POLA1, PRPS2, PDE7A, NUDT5, IMPDH2, POLD4, ADCY7, PRIM1, NT5E,*	2.04e-06
Peroxisome	*ACSL5, CAT, GSTK1, ABCD2, MPV17, PXMP2, PECR, HSD17B4, IDH1, ECH1*	3.46e-06
Pancreatic cancer	*RB1, RAC2, ARHGEF6, RALBP1, CDK4, IKBKB, TP53, MAPK1, CDK6*	1.06e-05
Chronic myeloid leukemia	*RB1, SOS1, CDK4, IKBKB, TP53, HDAC1, CBL, MAPK1, CDK6*	1.30e-05
Wnt signalling pathway	*PPP3CA, RAC2, PRKCB, TP53, NLK, GSK3B, PPP2R5B, PLCB2, DAAM1, ROCK2, CAMK2G, PRKACB*	2.00e-05
Chemokine signalling pathway	*RAC2, WAS, SOS1, CX3CR1, PRKCB, IKBKB, GSK3B, MAPK1, PLCB2, ROCK2, DOCK2, PRKACB, ADCY7*	3.56e-05
Apoptosis	*ATM, PPP3CA, DFFB, APAF1, DFFA, CASP10, IKBKB, PRKACB, TP53*	3.69e-05

Supplementary Figure 8 – Modules of seasonally co-regulated genes in the BABYDIET dataset
From the 6,359 seasonal probesets (mapping to 5,136 ENSMBL genes), 11 modules of co-regulated genes were generated. A full list of genes present in each module is shown in Extended Data Table 12. Pathway analysis of the genes in each module was performed using KEGG pathways, as described in the methods. The pathways with the strongest enrichment, and their gene members are shown, alongside their adjusted *P*-value.

Supplementary Figure 9

KEGG Pathway Analysis of seasonal adipose tissue genes

Pathway Name	#Genes	EntrezGene	Adj. P value
Metabolic pathways	92	1355 7360 4712 1798 3631 55902 64132 1854 5336 9061 9488 1312 2628 1786 55454 51703 124454 8813 10026 7378 48 535 63917 2585 3417 29926 10327 5557 7263 5445 5335 39 5050 51181 23530 56994 8992 3707 2584 549 3636 23556 4594 1718 6240 1327 128869 8702 5373 51004 374291 5281 1119 128 64768 3632 1603 79868 51227 3155 5833 8694 224 51144 5431 8803 2673 516 10678 669 6342 3295 9517 5286 56655 55361 847 159 5980 9296 50484 8613 2224 3614 6713 34 3145 498 9533 216 411 11285	P=8.69e-18
Pathways in cancer	40	2033 836 7184 2034 7157 5604 11186 27148 2535 7048 329 25 1387 3725 1399 5925 4193 1499 5728 5899 5336 1612 1027 10342 841 6774 5970 6655 7170 6654 5468 7428 867 2260 5595 5335 1398 3091 1487 54583	P=3.90e-13
Renal cell carcinoma	15	1399 2033 3725 1387 6655 2889 2034 1398 6654 3091 7428 201163 5604 5595 54583	P=3.54e-08
Chronic myeloid leukemia	15	25 1399 6655 5925 4193 1398 6654 7157 5604 1027 867 7048 5970 1487 5595	P=4.93e-08
ErbB signaling pathway	16	25 1399 3725 6655 817 5335 1398 6654 5336 5604 1027 6198 2002 867 818 5595	P=6.29e-08
Prostate cancer	16	2033 6655 7184 6654 7157 5604 2260 5595 1387 5925 4193 1499 5728 1027 80310 5970	P=7.41e-08
MAPK signaling pathway	27	6655 836 5530 6237 6654 6195 9448 7157 5604 8491 1846 2002 56940 7048 5495 9064 2260 5595 10235 3725 1399 2768 1398 11184 5494 5970 5567	P=2.25e-07
Neurotrophin signaling pathway	18	6655 2889 6654 10782 6195 7157 5604 5595 3725 1399 25 5335 817 1398 5336 5970 818 7529	P=2.99e-07
Regulation of actin cytoskeleton	23	6655 54961 23191 6237 54434 6654 8874 5604 3678 4627 55740 26999 2260 5595 1399 2768 2934 1398 5216 80310 81 7414 4636	P=5.80e-07
Ubiquitin mediated proteolysis	18	8924 23291 51433 7428 7332 867 329 9246 4193 55070 8925 7318 51343 51529 7322 26091 7326 10477	P=6.29e-07

Supplementary Figure 9 – Pathways amongst seasonal genes common to blood and adipose tissue
Between the BABYDIET and adipose datasets, 1,213 genes were found to be seasonal in both tissues. KEGG pathway analysis of these genes revealed various pathways to be shared between both tissues, notably metabolic processes and genes implicated in the risk to cancer. Pathway analysis was carried out as described in the materials and methods.
